# FIGO (International Federation of Gynecology and Obstetrics) initiative on fetal growth: Best practice advice for screening, diagnosis, and management of fetal growth restriction

**DOI:** 10.1002/ijgo.13522

**Published:** 2021-03-19

**Authors:** Nir Melamed, Ahmet Baschat, Yoav Yinon, Apostolos Athanasiadis, Federico Mecacci, Francesc Figueras, Vincenzo Berghella, Amala Nazareth, Muna Tahlak, H. David McIntyre, Fabrício Da Silva Costa, Anne B. Kihara, Eran Hadar, Fionnuala McAuliffe, Mark Hanson, Ronald C. Ma, Rachel Gooden, Eyal Sheiner, Anil Kapur, Hema Divakar, Diogo Ayres‐de‐Campos, Liran Hiersch, Liona C. Poon, John Kingdom, Roberto Romero, Moshe Hod

**Affiliations:** ^1^ Division of Maternal Fetal Medicine Department of Obstetrics and Gynecology Sunnybrook Health Sciences Centre University of Toronto Toronto ON Canada; ^2^ Center for Fetal Therapy Department of Gynecology and Obstetrics Johns Hopkins University Baltimore MD USA; ^3^ Fetal Medicine Unit Department of Obstetrics and Gynecology Sheba Medical Center Tel‐Hashomer Sackler Faculty of Medicine Tel‐Aviv University Tel Aviv Israel; ^4^ Third Department of Obstetrics and Gynecology Aristotle University of Thessaloniki Thessaloniki Greece; ^5^ Maternal Fetal Medicine Unit Division of Obstetrics and Gynecology Department of Biomedical, Experimental and Clinical Sciences University of Florence Florence Italy; ^6^ Maternal‐Fetal Medicine Department Barcelona Clinic Hospital University of Barcelona Barcelona Spain; ^7^ Division of Maternal‐Fetal Medicine Department of Obstetrics and Gynecology Thomas Jefferson University Philadelphia PA USA; ^8^ Jumeira Prime Healthcare Group Emirates Medical Association Dubai United Arab Emirates; ^9^ Latifa Hospital for Women and Children Dubai Health Authority Emirates Medical Association Mohammad Bin Rashid University for Medical Sciences, Dubai, United Arab Emirates; ^10^ Mater Research The University of Queensland Brisbane Qld Australia; ^11^ Department of Gynecology and Obstetrics Ribeirão Preto Medical School University of São Paulo Ribeirão Preto São Paulo Brazil; ^12^ African Federation of Obstetricians and Gynaecologists Khartoum Sudan; ^13^ Helen Schneider Hospital for Women Rabin Medical Center Petach Tikva Israel; ^14^ Sackler Faculty of Medicine Tel‐Aviv University Tel Aviv Israel; ^15^ UCD Perinatal Research Centre School of Medicine National Maternity Hospital University College Dublin Dublin Ireland; ^16^ Institute of Developmental Sciences University Hospital Southampton Southampton UK; ^17^ NIHR Southampton Biomedical Research Centre University of Southampton Southampton UK; ^18^ Department of Medicine and Therapeutics The Chinese University of Hong Kong Hong Kong SAR China; ^19^ Hong Kong Institute of Diabetes and Obesity The Chinese University of Hong Kong Hong Kong SAR China; ^20^ FIGO (International Federation of Gynecology and Obstetrics) London UK; ^21^ Soroka University Medical Center Ben‐Gurion University of the Negev Be’er‐Sheva Israel; ^22^ World Diabetes Foundation Bagsværd Denmark; ^23^ Divakars Speciality Hospital Bengaluru India; ^24^ Medical School and Santa Maria Hospital University of Lisbon Portugal; ^25^ Sourasky Medical Center and Sackler Faculty of Medicine Lis Maternity Hospital Tel Aviv University Tel Aviv Israel; ^26^ Department of Obstetrics and Gynecology Prince of Wales Hospital The Chinese University of Hong Kong Shatin Hong Kong SAR, China; ^27^ Division of Maternal Fetal Medicine Department of Obstetrics and Gynecology Mount Sinai Hospital University of Toronto Toronto ON Canada; ^28^ Perinatology Research Branch *Eunice Kennedy Shriver* National Institute of Child Health and Human Development National Institutes of Health U.S. Department of Health and Human Services Bethesda MD USA

**Keywords:** detection, diagnosis, Fetal growth restriction, FIGO initiative, management, monitoring

## CONTENTS

**Table:** 

1. Executive summary	7
2. Target audience	11
3. Assessment of quality of evidence and grading of strength of recommendations	12
4. Fetal growth restriction: background, definition, etiology, and risks	13
4.1. Background	13
4.2. Terminology and definitions	13
4.2.1. Consensus‐based definition of placenta‐related fetal growth restriction	13
4.2.2. Early‐ versus late‐onset fetal growth restriction	13
4.3. Etiology of fetal growth restriction	16
4.4. Risks associated with fetal growth restriction	16
4.5. Recommendations	17
5. Early prediction and prevention of fetal growth restriction	18
5.1. History‐based risk factors	18
5.2. Biochemical markers	18
5.3. Ultrasound markers	19
5.4. Prediction models	19
5.5. Prevention of fetal growth restriction in high‐risk populations	20
5.5.1. Lifestyle modifications	20
5.5.2. Medical interventions	20
5.6. Recommendations	21
6. Detection of fetal growth restriction	22
6.1. Symphysis–fundal height	22
6.2. Sonographic fetal weight estimation	22
6.3. Is there a role for routine third‐trimester ultrasound to assess fetal growth?	22
6.4. Which growth chart should be used to determine fetal weight percentile?	23
6.4.1. Growth references versus growth standards	23
6.4.2. Charts based on birth weight versus sonographic fetal weight estimation	23
6.4.3. Universal versus customized charts	24
6.4.4. Description of commonly available charts	24
6.4.5. How to choose the best chart	25
6.5. How to assess fetal growth in twin gestations	26
6.6. Recommendations	27
7. What kind of investigations should be performed when fetal growth restriction is suspected?	28
7.1. Detailed history	28
7.2. Detailed anatomy scan	28
7.3. Doppler studies	28
7.4. Additional testing	28
7.5. Recommendations	29
8. Management of pregnancies with fetal growth restriction	30
8.1. Monitoring	30
8.1.1. Fetal movement counting	30
8.1.2. Fetal heart rate monitoring	30
8.1.3. Computerized fetal heart rate monitoring	32
8.1.4. Ultrasound measurement of amniotic fluid volume	33
8.1.5. Biophysical profile scoring	33
8.1.6. Umbilical artery Doppler	33
8.1.7. Cerebral artery Doppler	33
8.1.8. Ductus venosus Doppler	34
8.1.9. Surveillance strategy	34
8.2. Timing of delivery	35
8.2.1. Gestational age‐related risks in fetal growth restriction	35
8.2.2. Gestational age‐related management strategy	35
8.2.3. Absolute delivery criteria for fetal growth restriction (independent of gestational age)	35
8.2.4. Relative delivery criteria for fetal growth restriction (adjusted for gestational age)	36
8.3. Mode of delivery and intrapartum considerations	36
8.4. Medical interventions	37
8.4.1. Antenatal corticosteroids	37
8.4.2. Magnesium sulfate for neuroprotection	37
8.4.3. Treatments under investigation	37
8.5. Recommendations	38
9. Postpartum assessment and counselling for future pregnancies	40
9.1. Infant follow‐up	40
9.2. Maternal follow‐up	40
9.3. Counselling regarding future pregnancies	40
9.3.1. Risk of recurrence based on severity and onset	40
9.3.2. Risk of recurrence based on placental histopathology	41
9.3.3. Role of thrombophilia screening	41
9.3.4. Preconception counselling and management of future pregnancies	41
9.4. Recommendations	43
10. Summary and future research directions	44
11. References	45

## 1 EXECUTIVE SUMMARY

Fetal growth restriction (FGR) is defined as the failure of the fetus to meet its growth potential due to a pathological factor, most commonly placental dysfunction. Worldwide, FGR is a leading cause of stillbirth, neonatal mortality, and short‐ and long‐term morbidity. Ongoing advances in clinical care, especially in definitions, diagnosis, and management of FGR, require efforts to effectively translate these changes to the wide range of obstetric care providers. This article highlights agreements based on current research in the diagnosis and management of FGR, and the areas that need more research to provide further clarification of recommendations.

The purpose of this article is to provide a comprehensive summary of available evidence along with practical recommendations concerning the care of pregnancies at risk of or complicated by FGR, with the overall goal to decrease the risk of stillbirth and neonatal mortality and morbidity associated with this condition. To achieve these goals, FIGO (the International Federation of Gynecology and Obstetrics) brought together international experts to review and summarize current knowledge of FGR.

This summary is directed at multiple stakeholders, including healthcare providers, healthcare delivery organizations and providers, FIGO member societies, and professional organizations. Recognizing the variation in the resources and expertise available for the management of FGR in different countries or regions, this article attempts to take into consideration the unique aspects of antenatal care in low‐resource settings (labelled “LRS” in the recommendations). This was achieved by collaboration with authors and FIGO member societies from low‐resource settings such as India, Sub‐Saharan Africa, the Middle East, and Latin America.

Aspects of FGR addressed in this article include prediction, diagnosis, investigation, management, and postpartum counselling. The main recommendations are given below and are summarized in Table 1 (section 8) and in the management algorithms for high‐resource settings (Figure 1a) and low‐resource settings (Figure 1b) (section 4).

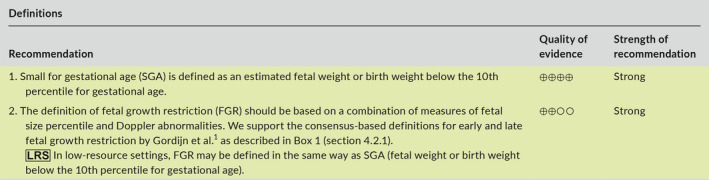

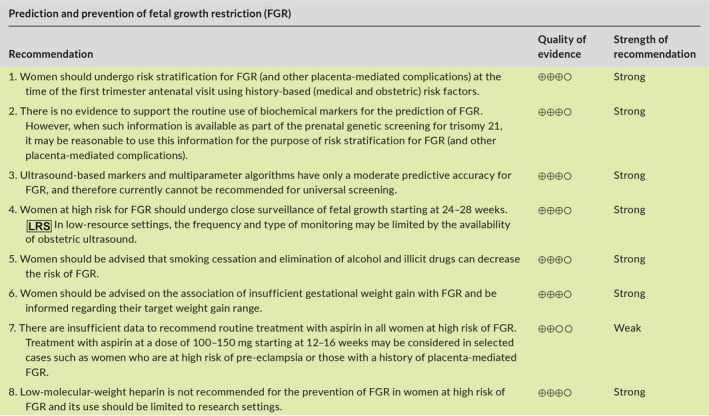

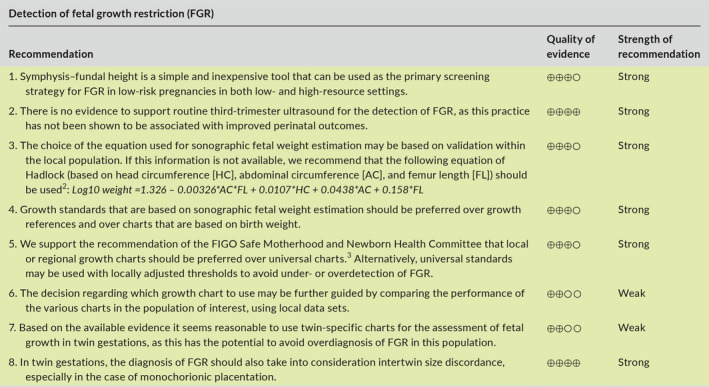

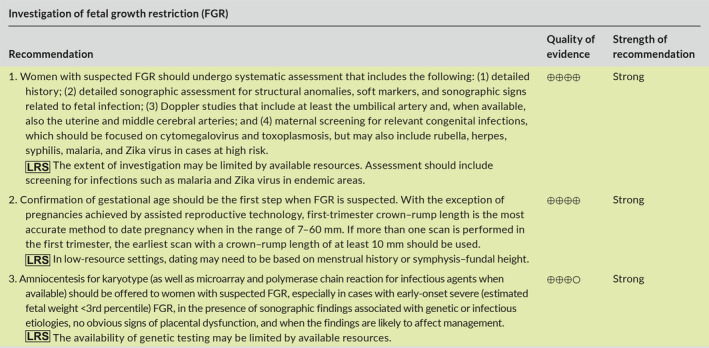

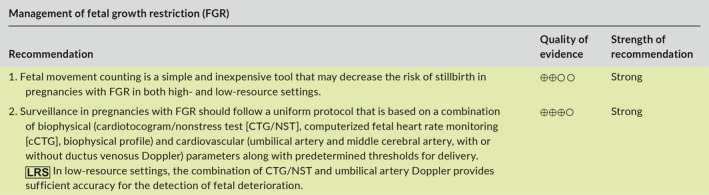

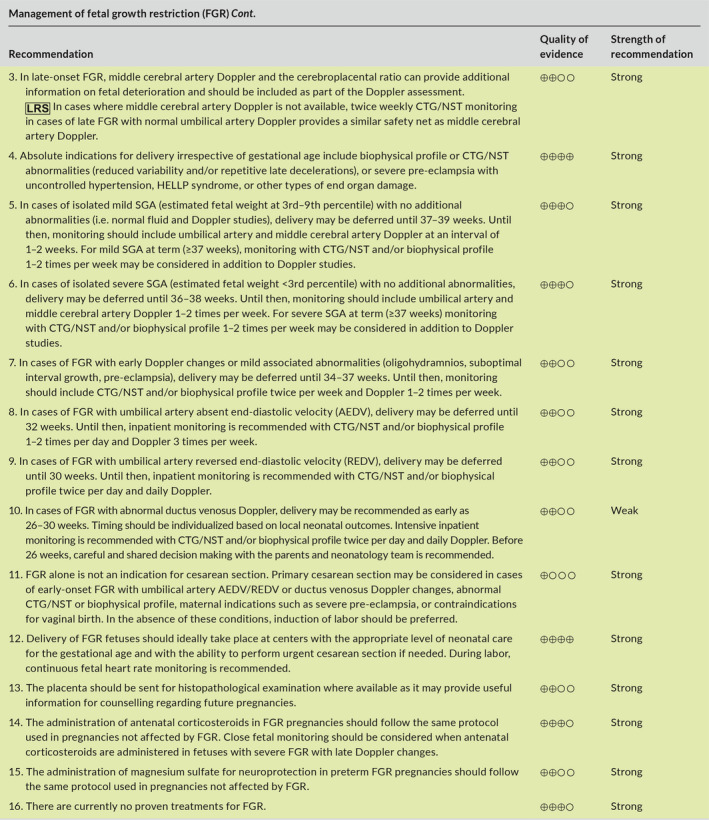

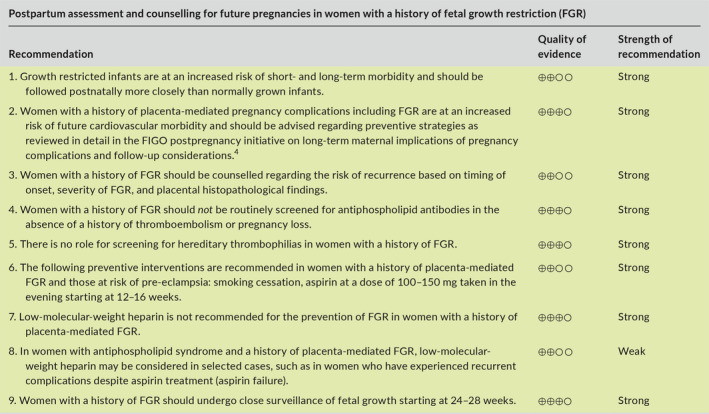

## 2 TARGET AUDIENCE

This article is directed at multiple stakeholders with the intention of bringing attention to the assessment of fetal growth, with a particular focus on the screening, diagnosis, and management of FGR, which is a leading cause of stillbirth and neonatal mortality and morbidity. This article proposes to standardize and provide guidance for the screening, prevention, diagnosis, and management of FGR.

The intended target audience includes:

**Healthcare providers**: all those qualified to care for pregnant women (obstetricians, maternal‐fetal medicine specialists, general practitioners, midwives, nurses, advance practice clinicians, radiologists, sonographers, pediatricians, and neonatologists).

**Healthcare delivery organizations and providers**: governments, federal and state legislators, healthcare management organizations, health insurance organizations, international development agencies, and nongovernmental organizations.

**Professional organizations**: international, regional, and national professional organizations of obstetricians and gynecologists, obstetric ultrasound, family practitioners, pediatricians, neonatologists, and worldwide national organizations dedicated to the care of pregnant women and their offspring.

## 3 ASSESSMENT OF QUALITY OF EVIDENCE AND GRADING OF STRENGTH OF RECOMMENDATIONS

In assessing the quality of evidence and grading of strength of recommendations, the article follows the terminology proposed by the Grading of Recommendations, Assessment, Development and Evaluation (GRADE) Working Group.^5^ This system uses consistent language and graphical descriptions for the strength and quality of the recommendations and the evidence on which they are based.

Recommendations are classified as strong or conditional (weak) (Table S1).^6^ The strength of recommendation is dependent not only on the quality of evidence, but also on factors such as risk–benefit, cost, resource allocation, values, and preferences. Thus, some recommendations may be based on low‐quality evidence but still represent a benefit that outweighs the risks and burdens, and therefore may be strongly recommended.

The overall quality of evidence was assessed for each of the recommendations and expressed using four levels of quality: very low, low, moderate, and high (Table S2).^7^ Considerations for quality of evidence include primarily the study design and methodology. As such, evidence based on randomized controlled trials is considered high‐quality evidence, observational studies provide moderate or low quality of evidence, and all others are very low. However, other parameters must be considered while assessing the level of evidence: risk of bias, study limitations, consistency of results, precision, publication bias, indirectness of evidence, and scarcity of evidence. For the quality of evidence, cross‐filled circles are used: ⊕○○○ denotes very low‐quality evidence; ⊕⊕○○ low quality; ⊕⊕⊕○ moderate quality; and ⊕⊕⊕⊕ high‐quality evidence.

## 4 FETAL GROWTH RESTRICTION: BACKGROUND, DEFINITION, ETIOLOGY, AND RISKS

### 4.1 Background

FGR is a common pregnancy complication that worldwide is a leading cause of stillbirth, neonatal mortality, and short‐ and long‐term neonatal morbidity.^8^, ^9^, ^10^, ^11^, ^12^, ^13^, ^14^, ^15^ The definition, diagnosis, and optimal management of FGR have generated controversy as clinicians strive for more harmonized care.

The purpose of this article is to provide a summary of the available evidence and provide recommendations regarding the early prediction and prevention, diagnosis, investigation, monitoring, and timing of delivery of pregnancies complicated by FGR, with the overall goal to decrease the risk of stillbirth and neonatal mortality and morbidity associated with this pregnancy complication. Given the variation in resources and expertise available for the assessment and monitoring of pregnancies complicated by FGR in different countries or regions, we have included, in addition to the standard of care or “best” recommendations, specific recommendations for low‐resource settings, which are marked as 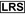 in the recommendation tables. Management algorithms for women in high‐resource and low‐resource settings are summarized in Figure 1a,b, respectively.

**FIGURE 1A:**
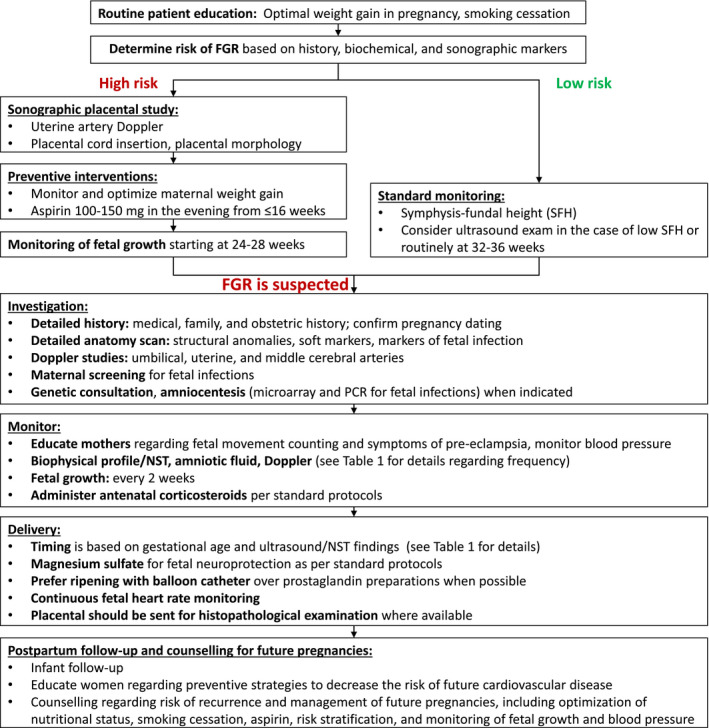
Approach to screening, diagnosis, and management of fetal growth restriction in high‐resource settings. Abbreviations: FGR, fetal growth restriction; NST, nonstress test; PCR, polymerase chain reaction; SFH, symphysis–fundal height.

**FIGURE 1B:**
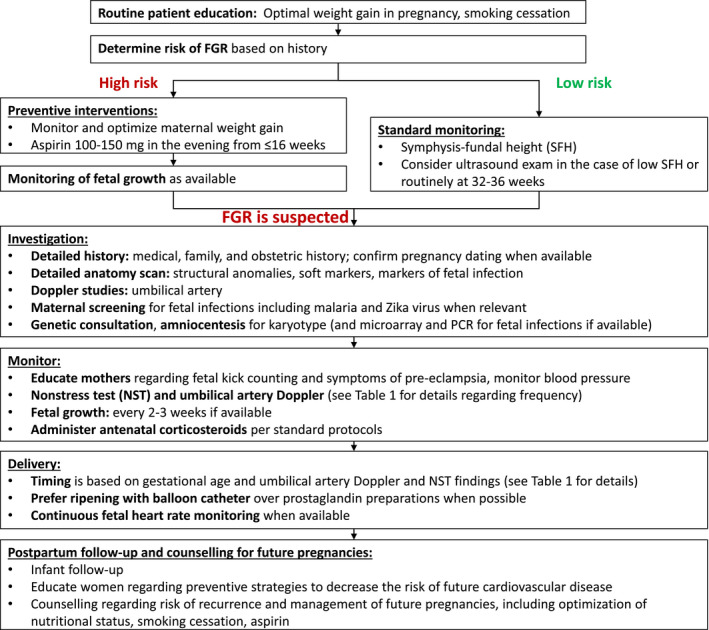
Approach to screening, diagnosis, and management of fetal growth restriction in low‐resource settings. Abbreviations: FGR, fetal growth restriction; NST, nonstress test; PCR, polymerase chain reaction; SFH, symphysis–fundal height.

### 4.2 Terminology and definitions

FGR is defined as the failure of the fetus to meet its growth potential due to a pathological factor, most commonly placental dysfunction. Clinically, this is reflected by a drop in fetal size percentiles over the course of gestation. However, fetal growth potential is difficult to determine, and serial assessments of fetal size to detect a drop in fetal weight percentile are usually not available. Instead, care providers most commonly have only a “snapshot” of fetal weight estimation at a given point in time. Therefore, in clinical practice, small for gestational age (SGA), defined as estimated fetal weight (EFW) or abdominal circumference below a certain threshold such as the 10th or 3rd percentile, is most commonly used to suspect FGR.

The use of SGA as a proxy for FGR has several limitations that need to be recognized. First, most SGA fetuses are constitutionally healthy small fetuses, whose smallness is merely the result of their predetermined growth potential (i.e. false‐positive diagnosis of FGR). Second, some growth‐restricted fetuses, depending on their original growth potential and timing of insult, may remain above the percentile threshold described above and may thus not be SGA (i.e. false‐negative diagnosis of FGR). Third, the use of SGA as a proxy for FGR is limited by the accuracy of sonographic fetal weight estimation, which has an estimation error of up to ±15%–20%. Finally, the diagnosis of SGA is highly dependent on the growth chart being used, which can therefore have a considerable effect on the proportion of fetuses or infants flagged as SGA in a given population.

It should be noted that there is inconsistency in the literature regarding the terminology described above, where some use the term FGR to describe a fetus with an estimated weight below the 10th percentile for gestational age and the term SGA to describe an infant with birth weight below the 10th percentile for gestational age. However, for the purpose of this article, the term SGA is used to indicate an EFW or birth weight below the 10th percentile for gestational age, and the term FGR to refer to a small fetus that has failed to achieve its growth potential because of a pathologic process.

#### 4.2.1 Consensus‐based definition of placenta‐related FGR

The major member societies of FIGO follow a definition using the 10th percentile as a means of diagnosing an SGA fetus, which then leads to further testing, assessment, and follow‐up. There are proposals to address the limitations of this definition, but their validity regarding reduction in adverse outcomes needs to be tested. For example, in an attempt to overcome some of the limitations described above, a consensus‐based definition for placenta‐mediated FGR has been proposed via a Delphi procedure.^1^ To decrease the likelihood of false‐positive and false‐negative diagnosis of FGR, the consensus definition was based on a combination of measures of fetal size (fetal weight estimation and abdominal circumference) and abnormal Doppler findings in the umbilical, uterine, and middle cerebral arteries, as described in Box 1. The implementation of this definition is limited by the lack of a recommendation on which growth chart should be used to define the 10th and 3rd percentiles for EFW and fetal abdominal circumference. In addition, further research is needed to correlate this definition with adverse perinatal outcomes.

Box 1 **Consensus‐based definitions for early and late fetal growth restriction.**

**Table:** 

Early‐onset FGR (<32 weeks) • EFW or AC <3rd percentile or • UA with AREDV or • EFW or AC <10th percentile, combined with one or more of the following: UA PI >95th percentile UtA PI >95th percentile	Late‐onset FGR (≥32 weeks) • EFW or AC <3rd percentile or • ≥2 of the following 3 criteria: EFW or AC <10th percentile EFW or AC crossing percentiles >2 quartiles on growth percentiles CPR <5th percentile or UA PI >95th percentile

Abbreviations: AC, fetal abdominal circumference; AREDV, absent or reversed end‐diastolic velocity; CPR, cerebroplacental ratio; EFW, estimated fetal weight; PI, pulsatility index; UA, umbilical artery; UtA, uterine artery. Adapted from Gordijn et al.^1^

#### 4.2.2 Early‐ versus late‐onset FGR

It has been suggested that FGR should be broadly classified, based on gestational age at the time of diagnosis, into early‐onset FGR (<32 weeks) and late‐onset FGR (≥32 weeks). The rationale underlying this classification is based on differences between these two phenotypes of FGR in severity, natural history, Doppler findings, association with hypertensive complications, placental findings, and management.^16^, ^17^, ^18^

Early‐onset FGR has a prevalence of 0.5%–1%, is usually more severe, and is more likely to be associated with abnormal umbilical artery Doppler than late‐onset FGR. The underlying placental pathology is frequently similar to that observed in cases of early‐onset pre‐eclampsia (maternal vascular malperfusion), which explains the strong association of early‐onset FGR with pre‐eclampsia. Therefore, early‐onset FGR is usually easier to detect, and the natural history tends to follow a predictable sequence of Doppler changes in the umbilical artery and ductus venosus. The main challenge in cases of early‐onset FGR is management (i.e. timing of delivery), by attempting to determine the optimal balance between the opposing risks of stillbirth and prematurity.^19^

Late‐onset FGR is more common than early‐onset FGR with a prevalence of 5%–10%. In contrast to early‐onset FGR, it is usually milder, is less likely to be associated with pre‐eclampsia, and is usually associated with normal umbilical artery Doppler. Therefore, the main challenge with regard to late‐onset FGR is diagnosis, while management (i.e. delivery) is relatively simple given that the diagnosis is commonly made during the late‐preterm or term periods, where the risks associated with delivery are relatively small. The diagnosis of late‐onset FGR mainly relies on adaptive changes in the cerebral circulation (“redistribution” or “brain‐sparing effect”), which is reflected by low resistance to flow in the middle cerebral artery thereby generating a low cerebroplacental ratio, as described in section 8.1.7. Given that the umbilical artery and ductus venosus Doppler studies are usually normal in cases of late‐onset FGR, the natural history in these cases is less predictable and there is a risk of sudden decompensation and stillbirth.^16^, ^19^

### 4.3 Etiology of fetal growth restriction

FGR is often the result of one or more maternal, placental, or fetal disorders that interfere with the normal mechanisms regulating fetal growth.^20^, ^21^ The most common etiologies of FGR are listed in Box 2. It is important to note that there is often confusion in the literature between “etiologies” (or pathogenetic pathways) and “risk factors” for FGR. For example, although maternal conditions such as chronic hypertension, kidney disease, systemic lupus erythematosus, and long‐standing diabetes are often listed as “maternal etiologies” for FGR, these conditions should probably be viewed instead as maternal risk factors for abnormal placentation that may result in placenta‐mediated FGR.

Box 2 **Common etiologies for fetal growth restriction.**

**Table:** 

Suboptimal uteroplacental perfusion and fetal nutrition Maternal (preplacental) factors • Hyposxemia (chronic lung disease, high altitude) • Anemia • Smoking, substance abuse (cocaine, methamphetamines) • Malabsorption, poor weight gain • Environmental toxins: air pollution, heavy metals (lead, mercury), perfluorooctanoic acid (PFOA) Placental factors • Maternal vascular malperfusion pathology (infarction, fibrin deposition, chronic abruption) • Fetal vascular malperfusion pathology • Chronic placental inflammation (e.g. villitis of unknown etiology) • Confined placental mosaicism Umbilical cord (postplacental) factors • Increased coiling • Increased cord length • True cord knot • Single umbilical artery • Marginal or velamentous cord insertion Fetal disorders Genetic disorders (chromosomal, micro deletions/duplications, single site mutations, epigenetic disorders) Structural anomalies (e.g. congenital heart disease, gastroschisis) Congenital infections (cytomegalovirus, toxoplasmosis, herpes, rubella, syphilis, Zika virus, malaria) Teratogen exposure (drugs, toxins)

Given that maternal nutrition and fetal growth are closely related,^22^, ^23^ maternal undernutrition is an important cause of FGR worldwide.^24^, ^25^, ^26^ The impact of maternal undernutrition on fetal growth depends on its timing and severity.^20^ To date, maternal interventions in dietary advice and modifications have lacked significant success in preventing FGR. While the mechanisms by which maternal anemia contribute to FGR are unclear, both impaired nutrient transport to the fetus ^27^ and abnormal placental adaptation to low maternal hemoglobin ^28^ have been suggested as potential mechanisms.

Abnormal placentation is a common cause of FGR, ^29^ which is often diagnosed by ultrasound Doppler studies ^30^ and typical histopathological placental findings.^31^, ^32^, ^33^

Chromosomal abnormalities have been suggested to contribute to up to 5% of FGR cases; triploidy and trisomy 13 and 18 are important considerations in early‐onset FGR and the risk of many aneuploidies is higher in the presence of structural fetal anomalies.^34^, ^35^, ^36^ In 1%–6% of cases of FGR with normal karyotype, submicroscopic (micro) duplications/deletions can be found using chromosomal microarray analysis,^35^ even when FGR is an apparently isolated finding.^37^ FGR is also more prevalent in fetuses with structural malformations, and the risk increases when multiple anomalies are present.^38^

FGR is related to intrauterine infection in up to 5% of cases.^20^, ^39^ Viral agents such as rubella, cytomegalovirus, HIV, and Zika are common causes of infection‐related FGR.^40^, ^41^, ^42^, ^43^, ^44^ Protozoan infections like toxoplasmosis and malaria are another important cause, especially in endemic areas.^45^, ^46^ The main mechanism involved in the pathogenesis of FGR in these cases is a decline in cell population.^20^ Finally, maternal exposure to teratogens such as radiation,^47^ illicit drugs,^48^, ^49^ and alcohol^50^ is another important etiology for FGR.

### 4.4 Risks associated with fetal growth restriction

The main short‐ and long‐term risks associated with FGR are listed in Box 3. It is associated with both fetal and obstetric complications. The most devastating complication is stillbirth,^51^, ^52^, ^53^ and there is a well‐established inverse relationship between weight percentile and the risk of stillbirth,^54^, ^55^, ^56^, ^57^ which is more pronounced in the early preterm period than at term.^58^ FGR is an important cause of iatrogenic preterm birth,^59^ as early delivery remains the main and perhaps only strategy for the prevention of stillbirth in cases of severe FGR.^16^, ^60^ FGR is also an independent risk factor for spontaneous preterm birth.^61^ Other obstetric complications associated with FGR include pre‐eclampsia and placental abruption, as the pathophysiology of these conditions is often closely related.^29^, ^30^, ^62^, ^63^, ^64^, ^65^, ^66^

Box 3 **Risks associated with fetal growth restriction.**

**Table:** 

Antenatal Stillbirth Pre‐eclampsia Placental abruption Preterm birth Neonatal (short term) Neonatal mortality Neonatal morbidity (hypoglycemia, hyperbilirubinemia, hypothermia, necrotizing enterocolitis, respiratory morbidity, intraventricular hemorrhage) Neonatal (long term) Neurodevelopmental disorders Metabolic syndrome (obesity, hypertension, diabetes, cardiovascular disease)

Despite ongoing improvements in neonatal care, FGR is associated with increased neonatal mortality and short‐term morbidity. The risk of perinatal mortality in term FGR is reported to be five‐ to 10‐fold higher than in appropriately grown neonates.^57^, ^61^, ^67^ The severity of FGR, Doppler abnormalities, and associated prematurity are independent predictors of neonatal complications.^68^ Among preterm infants, the co‐presence of FGR further increases the risk of certain prematurity‐related complications such as respiratory morbidity, intraventricular hemorrhage, necrotizing enterocolitis, and metabolic disorders.^57^ Among term infants, FGR increases the risks of low cord artery pH,^69^ low Apgar score,^69^ and neonatal complications such as hypoglycemia, hypothermia, and jaundice.^70^, ^71^, ^72^

Growth‐restricted infants are also at risk of long‐term complications including neurodevelopmental impairment ^11^, ^73^, ^74^, ^75^, ^76^, ^77^, ^78^ and noncommunicable diseases.^15^, ^79^, ^80^, ^81^, ^82^ This is discussed in greater detail in section 9.1 (Infant follow‐up).

### 4.5 Recommendations

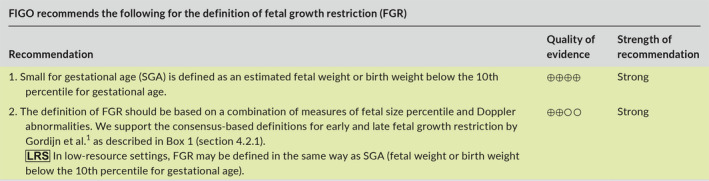

## 5 EARLY PREDICTION AND PREVENTION OF FETAL GROWTH RESTRICTION

Early prediction of FGR is important as it can identify women at high risk of FGR who may benefit from preventive interventions and close monitoring during pregnancy. Box 4 lists the most common risk factors for FGR. While the predictive value of individual risk factors is low, clinical prediction models that are based on combinations of the risk factors outlined below can considerably improve the prediction of FGR. One important limitation of most of the studies on early prediction of FGR is the lack of a gold standard for the antenatal or postnatal diagnosis of FGR. As such, there is wide variation among studies regarding the outcomes being predicted, including either SGA (birth weight below the 10th or 3rd percentile) or adverse perinatal outcomes that are associated with (but are not specific to) FGR. As many SGA infants are constitutionally small and healthy, differentiating between healthy small fetuses and those that are small due to FGR is critically important. As a rule, the prediction of early‐onset severe FGR is better than of late‐onset FGR.

Box 4 **Risk factors for fetal growth restriction.**

**Table:** 

History‐based risk factors Maternal demographics • Advanced age • Underweight • Living in high altitude • Severe anemia, hemoglobinopathies • Environmental factors (air pollution, heavy metals, heat) Medical conditions • Chronic hypertension • Chronic kidney disease • Systemic lupus erythematosus • Inflammatory bowel disease • Antiphospholipid syndrome • Pregestational diabetes (long standing) Obstetric history • Previous pregnancy affected by FGR or pre‐eclampsia Biochemical markers Low PlGF Low PAPP‐A High AFP Ultrasound‐based markers Uterine artery: pulsatility index >95th percentile Uterine artery: bilateral notching Marginal or velamentous cord insertion Two‐vessel cord (single umbilical artery) Abnormal placental morphologya Decreased fetal growth velocity

Abbreviations: FGR, fetal growth restriction; PlGF, placental growth factor; PAPP‐A, pregnancy‐associated plasma protein‐A; AFP, alpha‐fetoprotein.

^a^Refers to placental dimension (short‐based thick placenta) and texture (calcifications, echogenic cystic lesions).

### 5.1 History‐based risk factors

Several maternal factors influence fetal growth and the risk of FGR: advanced maternal age, racial/ethnic origin (e.g. South Asian), consanguinity, low body mass index, nulliparity, use of recreational drugs and alcohol, assisted reproductive technology, and medical disorders such as chronic hypertension, diabetes mellitus, and autoimmune conditions (Box 4).^83^, ^84^, ^85^, ^86^, ^87^, ^88^, ^89^ Cigarette smoking is a common risk factor for FGR and reduces birth weight by an average of 200 g in a dose–response manner.^90^ In a cohort of 33 602 pregnancies, maternal characteristics predicted 37% of women who subsequently delivered SGA neonates (birth weight <5th percentile) at a false‐positive rate of 10%.^83^

Some risk factors for FGR are especially relevant in low‐resource countries. In a recent review from Africa, the main risk factors reported were low maternal nutritional status, HIV infection, malaria, and hypertensive diseases. Based on these findings, the authors concluded that to a large extent FGR in Africa is preventable through established interventions for malaria, HIV, and maternal undernutrition.^42^ In addition, exposure during pregnancy and lactation to toxic environmental chemicals and heavy metals has become a growing problem, especially in low‐resource countries.^91^

### 5.2 Biochemical markers

At this point there is no role for routine screening with serum biomarkers for FGR. However, when biochemical markers are available as part of prenatal genetic screening for trisomy 21, it may be reasonable to use this information for the purpose of risk stratification for FGR.

The placenta releases multiple factors into maternal circulation from the early stages of pregnancy, and first‐trimester serum levels of some of these factors have been shown to be associated with subsequent placenta‐mediated complications.^92^, ^93^ Low levels of pregnancy‐associated plasma protein‐A (PAPP‐A), a placental glycoprotein produced by the syncytiotrophoblast layer, have been associated with adverse pregnancy outcomes including SGA. A meta‐analysis including 32 studies and 175 240 pregnancies found that PAPP‐A levels below the 5th percentile had a moderate association with birth weight below the 10th percentile (OR 2.08, positive predictive value of 18%), while the association was stronger for PAPP‐A levels below the 1st percentile (OR 3.4; positive predictive value of 28%).^94^ Thus, although women with low PAPP‐A are at increased risk for FGR, the majority of these women will have a normal pregnancy outcome, especially as an isolated biomarker in healthy women. However, a low PAPP‐A level is often considered an indication for closer monitoring of fetal growth.^95^ Elevated second‐trimester maternal serum levels of alpha‐fetoprotein are thought to reflect abnormal placental permeability and are associated with increased risk of placenta‐mediated complications including FGR and stillbirth.^96^, ^97^ The combination of low PAPP‐A in the first trimester and high alpha‐fetoprotein in the second trimester is particularly predictive of severe FGR.^98^ Elevated human chorionic gonadotropin (hCG) levels greater than 2.5 MoM in the second trimester, alone or combined with high alpha‐fetoprotein levels, are also associated with an increased risk of SGA.^99^

Angiogenic factors play a key role in the regulation of placental vascular development.^100^ Placental growth factor (PlGF) is a proangiogenic factor highly expressed in the syncytiotrophoblast and the maternal endothelium. Impaired placentation is associated with reduced placental production of this protein. Low first‐trimester PlGF levels have been shown to be associated with adverse pregnancy outcome including pre‐eclampsia and SGA.^101^, ^102^, ^103^, ^104^ In a case–control study of 296 pregnancies with SGA and 609 controls, the detection rate of low PlGF for SGA at a false‐positive rate of 5% and 10% was 15% and 21%, respectively. The combined use of PlGF and PAPP‐A increased the detection rate to 19% and 27%, respectively.^103^ A multicenter screening study found that the detection rate of a combined screening by maternal factors, fetal biometry, and serum PlGF and alpha‐fetoprotein at 19–24 weeks for the delivery of SGA infants below the 5th percentile at less than 32, 32–36, and greater than or equal to 37 weeks of gestation was 100%, 76%, and 38%, respectively, at a false‐positive rate of 10%.^96^

Findings are less consistent for soluble fms‐like tyrosine kinase‐1 (sFlt‐1), an antiangiogenic factor released from the placenta that results in maternal endothelial dysfunction characteristic of pre‐eclampsia.^105^ Although maternal serum sFlt‐1 levels are known to be elevated in pre‐eclamptic pregnancies, a large case–control study demonstrated that high levels of sFlt‐1 at 10–14 weeks were actually associated with a slightly reduced risk of SGA (OR 0.92; 95% CI, 0.88–0.96).^101^ Therefore, the sFlt‐1:PlGF ratio test used to diagnose pre‐eclampsia should not be used in the first trimester as a screening test for FGR.^106^

### 5.3 Ultrasound markers

Several ultrasound‐based markers have been shown to be predictive of FGR, including uterine artery Doppler, placental morphology, and placental volumes. However, given their modest predictive accuracy, they cannot be recommended for universal screening for FGR.

Increased uterine artery resistance largely reflects a failure of extravillous cytotrophoblast invasion and transformation of the spiral arteries and is associated with the development of pre‐eclampsia and FGR due to maternal vascular malperfusion of the placenta.^107^

First‐ and second‐trimester abnormal uterine artery Doppler waveforms, defined as mean pulsatility index above the 95th percentile, have been shown to be associated with FGR.^108^, ^109^, ^110^ In a large prospective cohort study of 4610 nulliparous women, uterine artery pulsatility index at 11^+0^ to 13^+6 ^weeks predicted 60% of preterm and 17% of term SGA infants at a false‐positive rate of 10%.^111^ Although uterine artery Doppler shows promise, especially for the prediction of early‐onset FGR, current evidence does not support routine screening with uterine artery Doppler for FGR in low‐ or high‐risk pregnancies.^112^

Sonographic evaluation of the placenta is a routine part of the obstetric ultrasound examination. A method for systematic two‐dimensional (2D) placental ultrasound examination has been described, often in combination with other parameters ^30^, ^113^, ^114^ Abnormal placental morphology is defined by placental dimensions, shape, texture, and cord insertion. Placental shape is considered abnormal when the placental thickness is above 4 cm or greater than 50% of placental length. Placental texture is defined as normal when it is homogenous, and abnormal when the placenta is heterogeneous and contains multiple echogenic cystic lesions or has a jelly‐like appearance with turbulent uteroplacental flow.^115^, ^116^ Placental cord insertion is defined as central (>2 cm from placental disc margin), marginal (within 2 cm of margin), or velamentous (inserting into the surrounding membranes).^114^ In a cohort of 60 high‐risk women with abnormal uterine artery Doppler, women with abnormal placental shape at 19–23 weeks had higher odds of FGR (OR 4.7) than women with normal placental shape.^108^ However, the use of 2D placental imaging has significant limitations, including difficulty in assessing nonanterior placentas and a wide variability in the morphology of normal placentas. Furthermore, there are no large‐scale prospective studies validating the use of this modality for prediction of FGR.^114^

Improvements in ultrasonographic imaging provide a tool for estimating placental volume using three‐ and four‐dimensional scanning techniques. Placental volume has been proposed as a marker for various obstetric complications related to defective placental function, including FGR.^117^, ^118^ A systematic review estimating the value of first‐trimester 3D placental volume for the prediction of SGA found a detection rate of 24.7% at a 10% false‐positive rate.^119^ Another parameter is the placental quotient, defined as the ratio of the placental volume to the fetal crown–rump length. The placental quotient was reported to have a high negative predictive value for perinatal complications but was not very useful when used for screening of SGA in a low‐risk population, with a sensitivity of 27.1%.^120^ The discriminatory ability of placental volume alone for SGA appears to be modest, but may be integrated into a multivariable screening model. However, the use of 3D placental volume as a routine screening tool for FGR is limited by the need for proper equipment and training required to obtain these measurements in a reproducible manner.

### 5.4 Prediction models

Currently there is no single screening test sufficiently predictive of FGR to recommend routine clinical use. Investigations are underway to combine various tests, but such prediction models have not been sufficiently validated in terms of outcomes studies and therefore must be considered investigative protocols at this time. In a prospective cohort of 4970 women, the combination of first‐trimester maternal serum PAPP‐A, beta hCG, maternal blood pressure, and uterine artery Doppler performed in the first trimester had a detection rate of 73% for early SGA (<34 weeks) but only 32% for late SGA (≥34 weeks).^19^ A different model that included maternal characteristics, first‐trimester blood pressure, uterine artery pulsatility index, PlGF, and sFlt‐1 was evaluated in a larger cohort of 9150 women and achieved a detection rate of 86% for early‐onset FGR and 66% for late‐onset FGR, both at a false‐positive rate of 10%.^19^, ^121^ In the second trimester, the SCOPE consortium examined 5606 healthy nulliparous women with singleton pregnancies and found that the combination of clinical risk factors, 15‐week biomarkers (53 biomarkers were used), and 20‐week ultrasound (fetal biometry and Doppler studies of the umbilical and uterine arteries) had only a moderate detection rate for SGA below the 10th percentile, with a positive predictive value of 32% and a negative predictive value of 91%.^122^

### 5.5 Prevention of fetal growth restriction in high‐risk populations

#### 5.5.1 Lifestyle modifications

Ideally, all women should plan their pregnancies, adopting a healthy lifestyle and optimizing any medical conditions and their body mass index. The preconception period provides an opportunity for health promotion with the aim of reducing accepted risk factors, including those associated with FGR.^123^

Insufficient gestational weight gain has been associated with an increased risk of FGR, especially in women with low body mass index (BMI, calculated as weight in kilograms divided by height in meters squared).^124^ Recognizing that these associations are only based on observational data, we still believe that it would be reasonable to recommend monitoring of weight gain and informing women of the target weight gain range, as recommended by the 2009 Institute of Medicine guidelines.^125^ These guidelines recommend a total gestational weight gain of 12.5–18 kg (28–40 lb) for underweight women (BMI <18.5); 11.5–16 kg (25–35 lb) for the normal weight group (BMI 18.5–24.9); 7–11.5 kg (15–25 lb) for overweight women (BMI 25.0–29.9); and 5–9 kg (11–20 lb) for obese women (BMI ≥30).^126^

Substance use, including smoking, alcohol, and illicit drugs, is associated with low birth weight and increased perinatal morbidity and mortality.^90^ Interventions to promote smoking cessation during pregnancy have been shown to result in a reduction in low birth weight (RR 0.81) and an increase in mean birth weight (+33 g).^127^ Women should be advised that smoking cessation at any point in gestation is of benefit, and that the greatest benefit is associated with cessation before 15 weeks of pregnancy.^128^ The risk of SGA with alcohol intake is increased with as little as one drink per day.^129^

#### 5.5.2 Medical interventions

Most studies on early prevention of placental complications have focused on pre‐eclampsia, with the results often being extrapolated to FGR due to the common pathophysiology. However, to date, other than lifestyle modifications, no medical interventions to prevent FGR have been clearly established.

Aspirin is recommended for women at increased risk of pre‐eclampsia, but there is some evidence that it may also reduce the risk of FGR ^130^, ^131^ In a recent meta‐analysis of 45 trials that included 20 909 women at high risk of pre‐eclampsia, the administration of aspirin starting at less than or equal to 16 weeks of pregnancy reduced the risk of FGR by nearly half (RR 0.56; 95% CI, 0.44–0.70), with higher dosages of aspirin associated with a greater reduction, favoring a dose of 100–150 mg.^132^ A second individual patient data meta‐analysis also supported earlier initiation of aspirin for the prevention of FGR, with an RR of 0.76 (95% CI, 0.61–0.94) for women randomized before 16 weeks versus an RR of 0.95 (95% CI, 0.84–1.08) for women randomized at 16 weeks or beyond.^131^ One randomized trial found that evening but not morning administration of aspirin is associated with reduction in the rate of pre‐eclampsia and FGR.^133^ However, it should be emphasized that most of the available data on aspirin come from studies that focused on the prevention of pre‐eclampsia as the primary outcome in women at high risk of pre‐eclampsia, with the prevention of FGR considered only as a secondary outcome. Furthermore, in the largest trial to date on the use of aspirin for the prevention of pre‐eclampsia (ASPRE trial), aspirin was not associated with a reduction in the risk of SGA below the 10th, 5th, or 3rd percentile.^130^ However, we believe that given the safety of aspirin and the overlap in the risk factors and pathogenesis of pre‐eclampsia and FGR, it is reasonable to recommend aspirin to women at high risk of FGR, using the same regimen of aspirin used for women at high risk of pre‐eclampsia. Most international guidelines recommend 100–150 mg aspirin to prevent FGR in women at high risk.^134^

The adjunct role of heparin in combination with aspirin to prevent placenta‐mediated complications in high‐risk situations was originally attributed to its anticoagulant properties and the speculative prevention of placental thrombosis. However, in vitro and in vivo data suggest heparins may have other biological properties including anti‐inflammatory, complement inhibition, and proangiogenic activities.^135^, ^136^, ^137^, ^138^ A study‐level meta‐analysis of six trials including 848 women showed that low‐molecular‐weight heparin (LMWH) was associated with a reduction in the composite outcome of pre‐eclampsia, birth weight below the 10th percentile, placental abruption, or pregnancy loss after 20 weeks (RR 0.52; 95% CI, 0.32–0.86) with similar risk reduction for SGA below the 10th and 5th percentiles. However, the higher‐quality trials suggest no treatment effect,^139^ and a subsequent individual patient data meta‐analysis looking at the same composite outcome found no beneficial effect of LMWH treatment (RR 0.64; 95% CI, 0.36–1.11).^140^ Likewise, the enoxaparin for pre‐eclampsia and intrauterine growth restriction (EPPI) trial included women at high risk for placenta‐mediated complications (with a high proportion of women with prior FGR) and showed no difference in the rate of the composite outcome (pre‐eclampsia or SGA <5th percentile) between treated and nontreated women.^141^ Therefore, based on the most up‐to‐date evidence, LMWH cannot be recommended for the prevention of FGR in women at high risk of placenta‐mediated complications. Its use for the prevention of FGR should therefore be limited to research settings, for example in women already on aspirin who are found to have abnormal levels of angiogenic markers prior to fetal viability.^142^

### 5.6 Recommendations

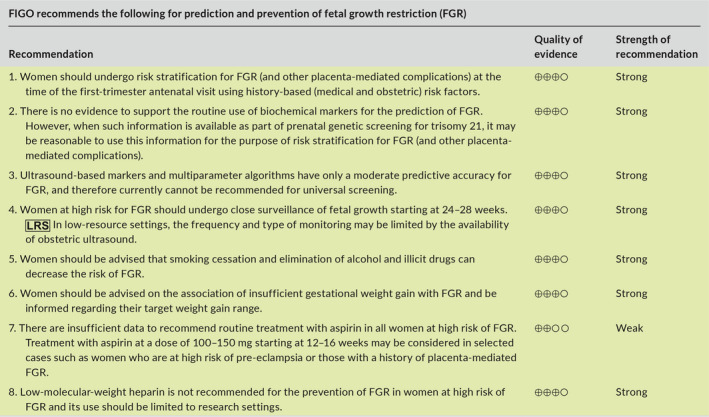

## 6 DETECTION OF FETAL GROWTH RESTRICTION

Detection of FGR is based on the identification of a fetus that is smaller than expected for gestational age, through either physical examination (symphysis–fundal height, SFH) or ultrasound.

### 6.1 Symphysis–fundal height

Measurement of SFH using a tape is a simple, inexpensive, and widely used strategy to screen for FGR.^143^, ^144^, ^145^, ^146^ SFH is measured with the woman in a supine position using a nonelastic metric tape after she has emptied her bladder. To decrease the interobserver variability, a standardized technique for measuring SFH should be followed.^144^, ^145^ SFH is defined as the distance from the upper border of the symphysis pubis bone to the top of the uterine fundus.^145^ SFH measured in centimeters between 24 and 38 weeks of gestation approximates the gestational age.^147^ Numerous local charts are currently used worldwide,^148^, ^149^, ^150^, ^151^, ^152^, ^153^, ^154^, ^155^, ^156^ with the recent addition of an international standard for SFH based on serial measurements.^145^ However, the accuracy of SFH measurement in predicting SGA (EFW <10th percentile) is limited, and there are no randomized controlled trials that compare SFH measurement with serial ultrasound evaluation of fetal biometry.^157^ In a meta‐analysis of 34 observational studies, SFH was reported to have a sensitivity of 58% and a specificity of 87% for predicting birth weight below the 10th percentile. There was marked heterogeneity between studies, mainly due to the use of different SFH charts.^158^ A single SFH measurement at 32–34 weeks of pregnancy has been reported to be approximately 65%–85% sensitive and 96% specific for detecting FGR.^143^ It is important to acknowledge that factors such as maternal obesity, uterine leiomyomas, and polyhydramnios may further limit the accuracy of SFH as a screening tool.^144^, ^159^

### 6.2 Sonographic fetal weight estimation

Sonographic fetal biometry is the cornerstone for detection of fetal growth disorders. Standard fetal biometry includes assessment of head circumference (HC), biparietal diameter, abdominal circumference (AC), and femur length (FL). Measurement of these biometric indices should be obtained by an experienced individual and in a standardized manner, as has been previously described.^160^ Fetal weight is estimated based on various combinations of the four biometric indices described above, using one of many published equations.^161^, ^162^, ^163^, ^164^, ^165^ The accuracy of most equations falls within the range of ±10%, and the error has been shown to be greater at the extremes of birth weight, and to be affected by factors such as fetal sex, presentation, and plurality (greater in twin gestations).^162^, ^163^, ^164^, ^166^, ^167^, ^168^, ^169^, ^170^, ^171^ Several studies have compared the accuracy of various equations. Most studies concluded that equations that are based on 3–4 biometric indices (rather than only 1–2 indices) provide the most consistent and accurate results. A recent systematic review ^165^ found that the Hadlock equation, based on three indices (HC, AC, and FL: Log10 weight = 1.326 − 0.00326*AC*FL + 0.0107*HC + 0.0438*AC + 0.158*FL), ^2^ provided the greatest accuracy. Since the accuracy of the various equations may vary between different populations, it may be reasonable for radiologists, sonographers, or care providers to choose an equation that has been validated within their local population and within the gestational age range in which it will be used. However, if such information is not available—a very frequent scenario—it seems reasonable to use the Hadlock equation as described above.

### 6.3 Is there a role for routine third‐trimester ultrasound to assess fetal growth?

In many countries, measurement of SFH is the primary screening tool for FGR in low‐risk pregnancies and ultrasound measurement of fetal biometry is performed only when indicated on the basis of risk factors or abnormal SFH.^134^, ^143^, ^172^, ^173^, ^174^ However, this approach fails to identify the majority of FGR infants,^146^ a concerning finding given that undetected FGR is associated with increased risk of adverse perinatal outcome and stillbirth.^53^, ^175^

An alternative approach is to perform a routine third‐trimester ultrasound for fetal weight estimation. However, a strategy for routine third‐trimester ultrasound in low‐risk pregnancies is not supported by available data and cannot be recommended.^176^, ^177^, ^178^

A meta‐analysis of 13 trials assessed the effect of routine sonographic weight estimation at more than 24 weeks of gestation on pregnancy outcomes in both unselected and low‐risk pregnancies.^178^ The authors found no association between routine sonographic EFW and adverse pregnancy outcomes including perinatal mortality, preterm birth, induction of labor, or cesarean section. In a recent randomized controlled trial of women with uncomplicated pregnancies, the use of serial (every 4 weeks) third‐trimester ultrasound was superior to routine care in the detection of a composite outcome of fetal growth or amniotic fluid abnormalities (RR 3.43; 95% CI, 1.64–7.17).^179^ However, it is important to note that the incidence of maternal or fetal morbidity was not significantly different between the groups. Similar results were reported by others.^180^ In contrast, the Pregnancy Outcome Prediction (POP) study prospectively assessed 3977 women and compared the detection of SGA (birth weight <10th percentile) by routine ultrasound versus clinically indicated ultrasound in the third trimester.^181^ The detection rate of SGA was nearly tripled in the routine ultrasound group (57% vs 20%). The risk of neonatal morbidity was increased only in the subset of SGA fetuses with fetal abdominal circumference growth velocity in the lowest decile (RR 3.9; 95% CI, 1.9–8.1), emphasizing the importance of combined analysis of fetal biometry and fetal growth velocity for better detection of fetuses at risk.^182^ Furthermore, it has been suggested that the prediction of FGR based on routine third‐trimester ultrasound can be improved by integrating EFW with additional biomarkers. A combined screening model that included maternal characteristics, third‐trimester EFW and placental Doppler, and biochemical markers (PlGF and estriol) achieved better performance than EFW alone in the detection of FGR (77% vs 64%) at a 10% false‐positive rate.^183^

There are many conceptual explanations to support third‐trimester ultrasound as it can assist in the diagnosis of clinically significant findings other than FGR, including fetal malpresentation,^184^ disorders of amniotic fluid, and fetal anomalies,^185^, ^186^ especially when combined with Doppler measurements and biochemical markers.^95^, ^187^, ^188^, ^189^ However, there is no evidence that this information improves outcomes when performed routinely in low‐risk pregnancies.

### 6.4 Which growth chart should be used to determine fetal weight percentile?

The interpretation of sonographic EFW depends on gestational age and is commonly classified as appropriate for gestational age, SGA, or large for gestational age, based on the calculation of EFW percentile using one of the many available growth charts. The choice of growth chart has been shown to have a considerable impact on the proportion of fetuses classified as either SGA or large for gestational age.^190^, ^191^ Over the past several years there has been an ongoing debate regarding the optimal growth chart that should be used, and numerous studies have compared the performance of a wide variety of charts in different populations with conflicting results. Prior to further discussion of specific charts, it is important to clarify the terminology and the types of charts that are currently available.

#### 6.4.1 Growth references versus growth standards

Growth references are descriptive charts that provide information on the distribution of weight of *all* newborns in a given population, and as such they include both normal and complicated pregnancies. Although growth references are useful as they provide information on the overall distribution of birth weight in the population, their use for the purpose of antenatal detection of FGR may be challenging as they are affected by the rate of pathologies in the population. For example, in populations with a high rate of large fetuses (e.g. due to a high rate of obesity and diabetes), the reference would be shifted upward. Similarly, in populations with a high rate of FGR (e.g. due to a high rate of malnutrition), the reference would be shifted downward.

For that reason, it may be reasonable to prefer growth standards over growth references for the antenatal detection of FGR. Growth standards are prescriptive charts that are based only on low‐risk or uncomplicated pregnancies, and as such provide information on what is the optimal fetal growth. There is variation between different growth standards with regard to the definition of “low‐risk” pregnancies; while some standards excluded women with pre‐existing medical conditions and pregnancy complications, others also excluded women below or above certain height or weight, women with suboptimal nutrition, low socioeconomic status, exposure to air pollution, high altitude etc. Since growth standards include only low‐risk uncomplicated pregnancies, their distribution is usually narrower (i.e. the 10th and 90th percentiles are closer to the mean) compared with growth references.

One important and practical aspect regarding the use of reference versus standard charts relates to the weight percentile threshold that should be used to trigger further evaluation for FGR. When using a growth reference, it is reasonable to use the 10th percentile for that purpose, as a considerable proportion of infants below the10th percentile will be affected by pathology. In the case of growth standard, however, using the same threshold of the 10th percentile would, per definition, identify 10% of the low‐risk pregnancies as suspected for FGR, which is not practical. Therefore, when using a growth standard, a lower threshold—such as the 5th or 3rd percentile—should be used to indicate further evaluation for FGR.

#### 6.4.2 Charts based on birth weight versus sonographic fetal weight estimation

**FIGURE 2:**
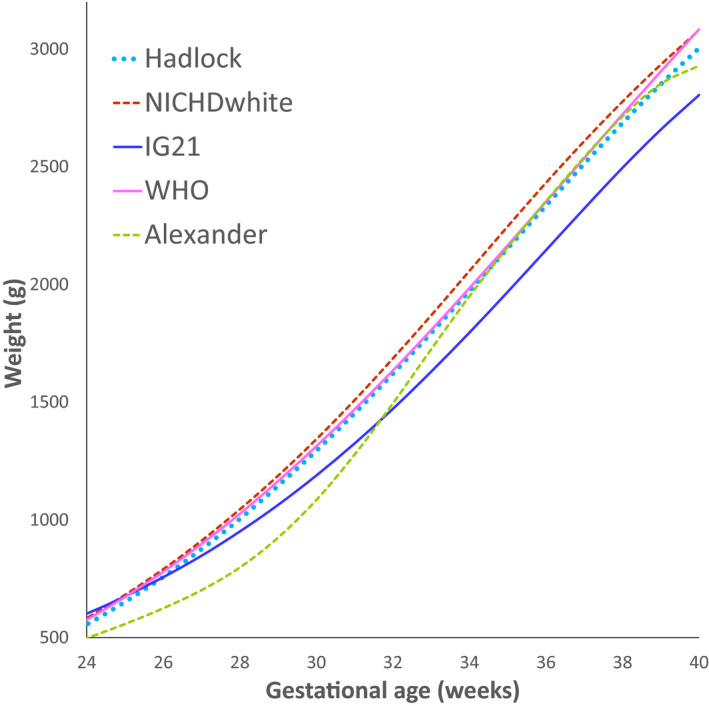
Comparison of the 10th percentile curves of common growth charts. Key: Hadlock: ultrasound‐based chart^193^; NICHD, National Institute of Child Health and Human Development chart^198^; IG21, Intergrowth‐21st chart^196^; WHO, World Health Organization chart^197^ Alexander: birth weight‐based chart.^192^ [Colour figure can be viewed at wileyonlinelibrary.com]

A second important distinction is between growth charts that are based on birth weight versus those that are based on sonographic EFW. Birth weight‐based charts rely on cross‐sectional data of infant birth weights across the full range of gestational ages, usually obtained from large databases. Different types of regression techniques are then used to calculate the mean and various percentiles of birth weight across gestation. These charts are commonly used as they are easy to develop. However, their main limitation is that infants born prematurely (before 37 weeks) are more likely to be affected by placental dysfunction and to be growth restricted. Therefore, these charts are likely to underestimate the optimal weight of fetuses during the preterm period, which in turn may lead to an underdiagnosis of FGR before 37 weeks. This is illustrated in Figure 2, where the birth weight‐based chart of Alexander (USA)^192^ is compared with several ultrasound‐based charts.

Therefore, it seems reasonable to prefer growth charts that are based on sonographic EFW over those that are based on birth weight. Ultrasound‐based growth charts are more difficult and expensive to develop, as they are usually based on data from prospective longitudinal studies where women undergo several sonographic weight estimations during pregnancy. However, these charts do not share the limitation of birth weight‐based charts, described above, and are thus more likely to reflect the optimal fetal growth throughout pregnancy (Figure 2). Another reason why ultrasound‐based charts should be preferred is that the measure used during pregnancy to assess fetal growth is sonographic EFW; it is therefore more appropriate to compare it to charts based on the same measure (i.e. sonographic EFW) rather than to charts based on birth weight. Some of the commonly used ultrasound based charts are presented in Figure 2.^193^

#### 6.4.3 Universal versus customized charts

One final distinction is between universal and customized growth charts, which represent a spectrum of approaches towards the similarity of the genetic growth potential of different fetuses across the world. At one end of this spectrum there are universal charts that are based on the assumption that under optimal conditions, all fetuses are expected to have the same growth potential, irrespective of their country of origin or race and that the only reason for the differences currently observed between different countries or races are purely due to environmental factors, such as malnutrition and environmental toxins. These ultrasound‐based charts are developed through multicenter, multinational, prospective longitudinal studies, where data on sonographic fetal growth from multiple countries are pooled into a single international universal chart. The best examples of such universal charts are the recently published Intergrowth‐21st^194^, ^195^, ^196^ and World Health Organization (WHO) charts.^197^

Others, however, believe that the variation in fetal growth between countries and races is not solely the result of environmental factors. Instead, it is suggested that genetic variation in growth potential contributes to the observed differences in fetal growth between race groups, and that race‐specific charts should therefore be preferred over universal charts. Examples of such race‐specific charts are the National Institute of Child Health and Human Development (NICHD) charts which include separate charts for white, black, Hispanic, and Asian women,^198^ and the recently published PRB/NICHD customized standard for African American women.^199^

According to the third approach, growth charts should be adjusted not only for maternal race but also for other physiologic factors that are thought to determine fetal growth potential, such as maternal height, weight, parity, and fetal sex. One such example is the Gestation Related Optimal Weight (GROW) software for customized growth percentiles.^200^, ^201^

At the other end of the spectrum is the individualized growth assessment (IGA) approach, which is based on estimation of the growth potential of the individual fetus, calculated from the second‐trimester growth velocity of that fetus. These estimates are used to generate individualized trajectories that are used to interpret fetal growth during the third trimester (https://igap.research.bcm.edu).^202^, ^203^, ^204^ While compelling, this approach requires earlier ultrasound exams during pregnancy, as well as appropriate software, and is therefore challenging at present for the purpose of FGR screening in the general population, and especially in low‐resource settings.

#### 6.4.4 Description of commonly available charts

The 10th percentile curves of some of the charts described above are compared in Figure 2. The Hadlock chart (1991), one of the most commonly used growth charts, is an ultrasound‐based standard. It is based on a cohort of 392 low‐risk, primarily white women from Texas. The Alexander chart (1996) is based on over 3 million singleton live births in the USA and is included as an example of a birth weight‐based reference to illustrate their limitation, which is the underestimation of optimal fetal growth during the preterm period.

The goal of the Intergrowth‐21st project (2014) was to develop a universal ultrasound‐based prescriptive growth chart. This was a prospective longitudinal study of 4321 low‐risk women from eight centers located in eight high‐ and middle‐income countries.^194^ The study had strict inclusion and exclusion criteria to ensure that participants were not exposed to environmental factors known to affect fetal growth, and it therefore aimed to reflect optimal fetal growth. Based on predetermined criteria, the authors concluded that the differences between participants from different countries in measures of skeletal growth (crown–rump length and head circumference) were similar enough to justify pooling the data, and they therefore generated a single universal chart. No information was provided on the differences between countries with respect to measures such as fetal weight estimation and abdominal circumference, which are used in clinical practice to detect FGR and are known to be associated with adverse perinatal outcomes. Interestingly, there were considerable differences in birth weight between infants from different countries, even in this highly selected group of women free from the negative influence of environmental factors known to affect fetal growth. For example, the mean birth weight at term in India was 2.9 kg, which was approximately 600 g lower than the mean birth weight in the UK (3.5 kg).^195^ These differences have led some to question the validity of the pooled chart and of the hypothesis that underlies the Intergrowth‐21st project.^205^, ^206^ As demonstrated in Figure 2, the 10th percentile of the Intergrowth‐21st chart is significantly lower throughout gestation than most other ultrasound‐based standards.

At around the same time, the results of the NICHD growth study (2015) were published.^198^ The overall design of this study was similar to that of the Intergrowth‐21st study. It was a prospective longitudinal study of 2334 low‐risk women from 12 centers in the USA. The authors found substantial differences in fetal weight between different race groups, and therefore developed separate race‐specific growth charts for white, black, Hispanic, and Asian women. The 10th percentile of the NICHD growth chart for white women is included as an example in Figure 2.

The WHO fetal growth charts were published in 2017.^197^ Similar to the Intergrowth‐21st project, this study aimed to develop a prescriptive universal chart to extend the previously published WHO child growth standard ^207^ to the fetal period. The design of this study was also similar to that of Intergrowth‐21st—a prospective longitudinal study of 1387 low‐risk women from 10 centers in 10 high‐ and middle‐income countries. Despite this, the results of the WHO study differed from those of Intergrowth‐21st in two aspects. First, the 10th percentile of the WHO chart is considerably higher than the Intergrowth‐21st chart, and is in fact almost identical to the 10th percentile of the Hadlock standard (Figure 2). Second, unlike Intergrowth‐21st, the investigators of the WHO study found substantial differences in fetal growth between the various countries, and concluded that “…populations, even under optimal nutritional conditions and environment, vary and that fetal growth varies and should be considered when the WHO fetal growth charts or any growth references are applied”.^208^ They expressed concern that use of a universal chart carries a risk of misclassification of FGR,^209^, ^210^, ^211^ and recommended that their chart should be adjusted in each country to the local population.

The benefit of customized charts remains a matter of debate. The GROW software incorporates certain factors that are believed to determine fetal growth potential (maternal race, height, weight, parity, and fetal sex) to calculate the predicted optimal (customized) weight at 40 weeks for each individual fetus.^200^, ^201^ The customized fetal growth curve is then determined retrospectively, based on a proportionality growth function derived from the ultrasound‐based Hadlock standard.^193^ The use of customized charts is appealing, especially in the setting of ethnically mixed populations where their use has been shown to decrease over‐ and underestimation of FGR rates in certain race groups.^212^ A large number of studies investigated the association of customized charts with adverse pregnancy outcomes compared with other birth weight‐ and ultrasound‐based charts, with conflicting results. Several studies found that customized charts performed better at predicting stillbirth and adverse neonatal outcomes,^66^, ^201^, ^211^, ^213^, ^214^, ^215^, ^216^ while others found no benefit and concluded that the benefit reported by others is merely because they are based on an ultrasound‐based chart and are thus more likely to reflect optimal fetal growth, while the act of customization has a minimal contribution to the stronger association with adverse outcome.^217^, ^218^, ^219^ Another criticism is that the GROW approach assumes that all fetuses follow the same growth trajectory (which is derived from the Hadlock chart)—an assumption that may not be true. Finally, it has been suggested that the required adjustment for multiple factors may be too complex for low‐resource countries and, in that setting, a simple adjustment to only one factor—mean birth weight at 40 weeks in the local population—is as predictive for adverse perinatal outcomes as the fully customized GROW charts.^220^ It may thus be reasonable for care providers to compare the performance of customized growth charts in their population with that of noncustomized charts (as discussed below), especially in regions or countries with a mixed population where the benefit of customization is expected to be greatest.

#### 6.4.5 How to choose the best chart

The conflicting results and conclusions regarding the growth charts described above have led to an ongoing debate about the best approach (i.e. universal versus customized charts), as well as to considerable confusion among care providers over which chart they should be using in their local population.

The FIGO Safe Motherhood and Newborn Health Committee recently published a position paper on the choice of reference charts for fetal growth and size at birth.^3^ In that paper, the committee reviewed in detail the commonly available charts and the available data on their predictive accuracy. The main conclusions were as follows: (1) local or regional charts are likely to be best to identify the 10th percentile of infants at highest risk, given that universal charts such as Intergrowth‐21st are likely to under detect SGA fetuses in high‐resource countries and, at the same time, over detect SGA in low‐ and middle‐income countries; (2) as an alternative, universal standards such as Intergorwth‐21st and WHO may be used with locally adjusted thresholds (e.g. 3rd or 5th percentile in low‐ or middle‐income countries versus 15th or 20th percentile in high‐income countries) to avoid under or overdetection of SGA; and (3) when assessing fetal size antenatally by ultrasound, fetal (i.e. ultrasound‐based) charts should be used rather than birth weight‐based charts. We fully endorse and support these recommendations.

**FIGURE 3:**
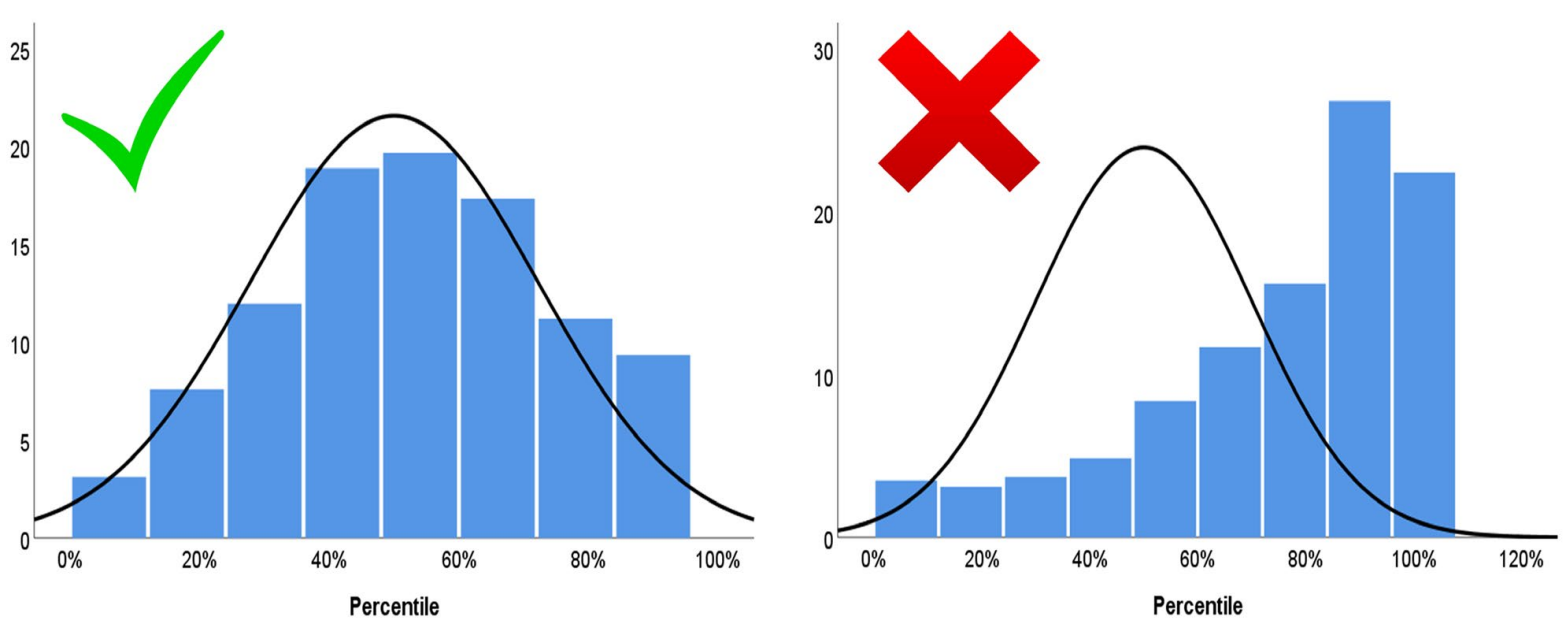
Illustration of the statistical validation of two charts in a local population. The left chart shows a good match to the population of interest: the distribution of fetal weight percentiles based on this chart follows a normal distribution that is centered at the 50th percentile, with approximately 10% of the population below the 10th and above the 90th percentile. The right chart shows a poor fit for the population of interest as it is skewed to the right: it overdiagnoses fetuses as large for gestational age and underdiagnoses small‐for‐gestational‐age fetuses. [Colour figure can be viewed at wileyonlinelibrary.com]

**FIGURE 4:**
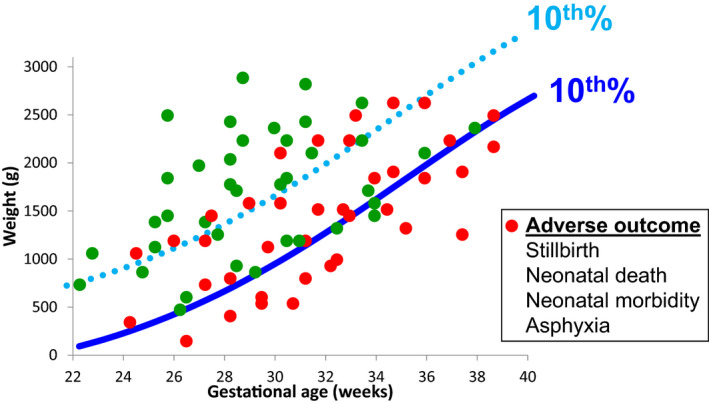
Illustration of the impact of the growth chart chosen on the trade‐off between detection rate and false‐positive rate of fetuses at risk of adverse outcome. Charts that are shifted upward (light blue dotted line) will have a higher detection rate for pregnancies at risk of adverse outcomes (red circles) but would also have a higher false‐positive rate (i.e. identify normal pregnancies [green circle] as being at risk). In contrast, charts that are shifted downward (dark blue solid line) will have a lower false‐positive rate (i.e. identify fewer normal pregnancies [green circle] as being at risk) but will also have a lower detection rate for pregnancies at risk of adverse outcomes (red circles). [Colour figure can be viewed at wileyonlinelibrary.com]

Furthermore, we as well as others,^160^, ^208^ believe that the decision on which chart to use can be further based on a comparison of performance of the various charts in the population of interest, using a local data set. This can be achieved by the following approaches: (1) statistical validation: finding the chart that matches best the distribution of fetal weight in low‐risk pregnancies in the local population. That is, identifying the chart that when applied to the local population yields weight percentiles that follow a normal distribution centered at approximately the 50th percentile, and identifies approximately 10% of the low‐risk population as being below the 10th percentile and above the 90th percentile, and approximately 5% of the population as being below the 5th percentile and above the 95th percentile. An example of this approach is provided in Figure 3; (2) outcome‐based validation: finding the chart for which the diagnosis of SGA has the best predictive value for adverse outcomes related to FGR..^211^, ^221^ While this approach seems compelling, interpretation of the predictive value of the different charts for adverse outcomes may be challenging, as there is a trade‐off between detection rate and false positive rate for adverse outcomes.^221^ Thus, charts that are shifted upward (e.g. Hadlock, WHO) would have a higher detection rate but also a high false‐positive rate, while charts that are shifted downward (e.g. Intergrowth‐21st) would have a lower false‐positive rate but would also have a lower detection rate for SGA fetuses at risk of adverse outcomes (Figure 4). Finding the chart that provides the best balance between these two measures requires careful consideration and should be based on a clear definition of the goals of screening.

### 6.5 How to assess fetal growth in twin gestations

Twin fetuses grow more slowly than singletons, starting from 28–32 weeks of gestation onward.^222^, ^223^, ^224^, ^225^ At term, approximately 30%–50% of twins would be identified as SGA (EFW <10th percentile) using singleton growth standards.^223^, ^226^, ^227^ The mechanisms underlying the relative smallness of twins remain unclear. While some believe that this represents a pathological phenomenon due to failure of the uteroplacental circulation to meet the demands of two fetuses (i.e. twins are more likely to be growth restricted due to the same mechanism responsible for FGR in singletons),^224^, ^228^, ^229^, ^230^ others suggest that this represents an early benign physiological adaptation of twins to the “crowded” intrauterine environment in an effort to delay the onset of labor (by decreasing uterine distension) and gain maturation at the expense of size.^231^ One important implication of this question relates to the growth standard that should be used in twins. If the slower growth of twins represents FGR, it would be reasonable to use singleton growth standards to identify the small twin fetus that, like SGA singletons, may be at increased risk for perinatal mortality and morbidity. However, if the relative smallness of twins is due to a benign adaptive mechanism, it may be preferable to use twin‐specific growth charts ^223^, ^232^, ^233^, ^234^, ^235^ to avoid overdiagnosis of FGR in twin gestations,^63^, ^227^ which is associated with increased use of resources, ultrasound exams, interventions, and patient anxiety.

Most current guidelines do not provide clear recommendations as to which type of charts should be used to monitor the growth of twins,^143^, ^236^, ^237^ while other guidelines specifically recommend the use of singleton‐based charts ^238^ or twin‐specific charts.^239^ As a result, singleton‐based standards are used by default in most centers to assess the growth of twins. However, recent data provide support to the hypothesis that the relative smallness of twins is a benign adaptive mechanism and, therefore, for the use of twin‐specific charts. For example, several studies suggest that the slower growth of twins is the result of differences in programming that is determined as early as the first trimester..^229^, ^240^, ^241^, ^242^, ^243^ In addition, it was found that the use of twin‐specific (versus singleton‐based) charts was associated with a marked decrease in the rate of twins classified as SGA, without affecting the detection rate of stillbirth, suggesting that twin‐specific charts can be used safely.^227^, ^244^, ^245^ Similar findings were reported in studies that investigated the association between the type of chart used (twin versus singleton charts) and other outcomes such as perinatal complications and long‐term morbidity.^246^, ^247^ Studies that investigated placental pathology findings reported that SGA twins (based on singleton charts) are less likely to have placental histopathological evidence of placental insufficiency when compared with SGA singletons.^248^, ^249^ In another recent study on the association between SGA and pre‐eclampsia, it was found that in contrast to singletons, the diagnosis of SGA in twins based on singleton charts was not associated with a greater risk of pre‐eclampsia, while the association of SGA in twins diagnosed using twin‐specific charts had the same magnitude of association with pre‐eclampsia to that observed between SGA and pre‐eclampsia in singletons.^63^ Overall, these findings provide support to the hypothesis that the relative smallness of twins is less likely to be the result of placental insufficiency and, thus, less likely to reflect true growth restriction. Based on that, we believe that it seems reasonable to use twin‐specific charts for the assessment of fetal growth in twin gestations, as this has the potential to avoid overdiagnosis of FGR and the consequences associated with this diagnosis.^246^ This approach is supported by the recent guidelines of the International Society of Ultrasound in Obstetrics and Gynecology (ISUOG).^239^ Of note, the diagnosis of FGR in twin gestations should also take into consideration intertwin size discordance, especially in the case of monochorionic placentation.^250^

### 6.6 Recommendations

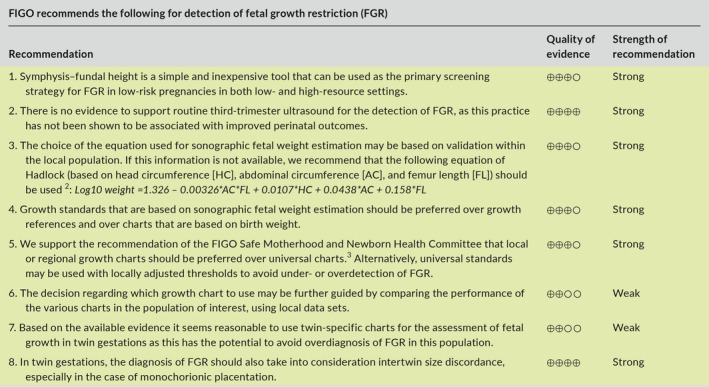

## 7 WHAT KIND OF INVESTIGATIONS SHOULD BE PERFORMED WHEN FETAL GROWTH RESTRICTION IS SUSPECTED?

Once FGR is suspected, a systematic investigation should be performed aimed at identifying the underlying etiology for fetal smallness, with the most important reasons being constitutional SGA, placental dysfunction, and fetal conditions such as genetic or infectious disorders. Establishing the most likely etiology is essential to allow for proper counseling, surveillance, and interventions. The investigation should consist of detailed history, evaluation of screening test results for trisomy 21 and biochemical markers, detailed sonographic assessment for structural anomalies and Doppler studies, and additional testing directed at genetic or infectious etiologies when they are suspected.

### 7.1 Detailed history

A detailed maternal and family history is essential to correctly identify the etiology of FGR. This should include information on maternal age, racial/ethnic group, height and weight, nutritional status, socioeconomic status, medications, cigarette smoking and use of recreational drugs, chronic medical conditions, personal or family history suggestive of thrombophilia, genetic disorders or consanguinity, obstetric history including birth weight of previous children, and confirmation of pregnancy dating by first‐trimester ultrasound.^143^

Advanced maternal age has been associated with FGR, with risk increasing for women over the age of 35 years.^251^, ^252^ Maternal social issues, low income, and domestic violence during pregnancy have been shown to be associated with low birth weight.^253^, ^254^ Poor nutritional status due to conditions such as celiac disease ^255^ and eating disorders is a potentially treatable cause of FGR.^256^, ^257^ Maternal smoking is an important and potentially modifiable risk factor for FGR.^258^, ^259^

History should also address the risk of congenital fetal infection with cytomegalovirus, toxoplasmosis, syphilis, Zika virus, and varicella‐zoster virus. Relevant questions include a history of febrile disease or rash in pregnancy or the periconceptional period, recent travel history to endemic areas (e.g. for Zika virus), and frequent exposure to young children (cytomegalovirus) or to domestic animals (toxoplasmosis).

Accurate dating of pregnancy is essential for the correct interpretation of estimated fetal size and to avoid a false diagnosis of FGR. Determining gestational age based on menstrual history is often unreliable.^260^, ^261^ Therefore, with the exception of pregnancies achieved by assisted reproductive technology, the crown–rump length measured at the time of first‐trimester ultrasound is the most accurate method to date pregnancy, and establishes gestational age with a precision of 5 days in 95% of cases.^262^, ^263^, ^264^, ^265^ Crown–rump length is most accurate for the purpose of dating when in the range of 7–60 mm.^266^, ^267^ Therefore, confirmation of gestational age based on first‐trimester ultrasound (when available) should be the first step when FGR is suspected. If more than one scan is performed in the first trimester, the earliest scan with a crown–rump length of at least 10 mm should be used.^268^

### 7.2 Detailed anatomy scan

Detailed anatomy scan should be routinely performed when FGR is suspected, especially in cases of early‐onset severe FGR. The presence of major structural anomalies, soft sonographic markers, or disorders of amniotic fluid (e.g. polyhydramnios) may raise the possibility of chromosomal, subchromosomal, or single gene abnormalities as the cause of FGR.^269^, ^270^ The presence of very shortened fetal long bones (shorter than –2SD and especially –4SD below the mean) should raise the possibility of skeletal dysplasia and indicates targeted genetic assessment.^271^, ^272^, ^273^ Attention should also be given to findings that are associated with congenital infections, especially in women with a relevant history, as described above. Examples of such sonographic findings include small head circumference, ventriculomegaly, brain or liver calcifications, periventricular hyperechogenicity, cortical brain malformations, echogenic bowel, hydrops, or placentomegaly.^40^, ^274^

### 7.3 Doppler studies

Doppler assessment is an integral part of the diagnostic process and management of FGR. The presence of abnormal Doppler findings in the uterine, umbilical, or middle cerebral arteries is highly suggestive of placental dysfunction as the underlying etiology of FGR. A more detailed description of the different types of Dopplers studies and their application in monitoring and timing of delivery in pregnancies complicated by FGR is provided in section 8 (Management of FGR).

It should be noted that umbilical artery Doppler findings may be normal in the early stages of placental FGR. Therefore, normal umbilical artery Doppler studies do not rule out placental dysfunction, and therefore serial monitoring is recommended in all cases of suspected FGR.^275^, ^276^ At the same time, abnormal umbilical artery Doppler is not pathognomonic of placental dysfunction, as certain genetic conditions (e.g. triploidy) may mimic early‐onset placental FGR, including the presence of abnormal umbilical artery Doppler, most likely due to concomitant placental insufficiency secondary to the abnormal placental karyotype.^34^, ^277^, ^278^, ^279^ In contrast to umbilical artery Doppler, uterine artery Doppler is less likely to be abnormal among fetuses with FGR and abnormal karyotype, and should therefore be considered to be more specific for primary placental FGR, especially in the presence of abnormal angiogenic markers in maternal blood.^34^, ^107^, ^280^

### 7.4 Additional testing

Screening for congenital infections should be offered when FGR is suspected, especially in cases of early‐onset FGR or when infection is possible based on history of ultrasound findings. Testing should be focused on cytomegalovirus and toxoplasmosis, but may also include rubella, varicella, and syphilis in cases at high risk for these infections. Testing for Zika virus and malaria should also be considered in the relevant travel history or location context. However, it should be noted that interpretation of serology results may be challenging due to limited specificity and cross‐reactivity of some of the assays, especially when baseline serology results prior to pregnancy or from early pregnancy are not available.^281^ When fetal infection is highly suspected based on serology results or clinical findings, further testing should be offered by means of amniocentesis for the detection of viral DNA in the amniotic fluid using polymerase chain reaction. In these cases, amniocentesis should be delayed until after 21 weeks of gestation and at least 6–8 weeks following the estimated onset of maternal infection to minimize the risk of false‐negative results.^274^, ^282^

Genetic consultation and genetic testing by amniocentesis should be offered to women with FGR, especially in cases of early‐onset or severe FGR (<3rd percentile), co‐presence of sonographic findings (such as structural anomalies, soft markers, or polyhydramnios), and the absence of obvious signs of placental dysfunction such as abnormal uterine or umbilical artery Doppler. In addition, women should be counselled about the risk of a genetic etiology even in the presence of “isolated” FGR (i.e. without associated fetal anomalies).^37^, ^270^, ^283^, ^284^, ^285^ A recent meta‐analysis of 10 studies found that in cases of isolated FGR, chromosomal microarray had an incremental yield of 4% (95% CI, 1%–6%) over karyotyping: 17 of 376 fetuses with isolated FGR and normal karyotype had significant findings in microarray, most commonly 22q11.2 duplication, Xp22.3 deletion, and 7q11.23 deletion. The incremental yield of microarray over karyotyping was even higher at 10% (95% CI, 6%–14%) in the presence of associated fetal malformations.^35^ Based on these data, it seems reasonable to offer amniocentesis with karyotype and microarray analysis (when available) to women with FGR, with the decision based on factors such as ultrasound findings, gestational age, lack of evidence of placental dysfunction, and whether the results of the amniocentesis would affect management. These data also suggest that the temptation to substitute amniocentesis by noninvasive prenatal testing (NIPT) using cell‐free fetal DNA analysis in this context should be strongly resisted.

### 7.5 Recommendations

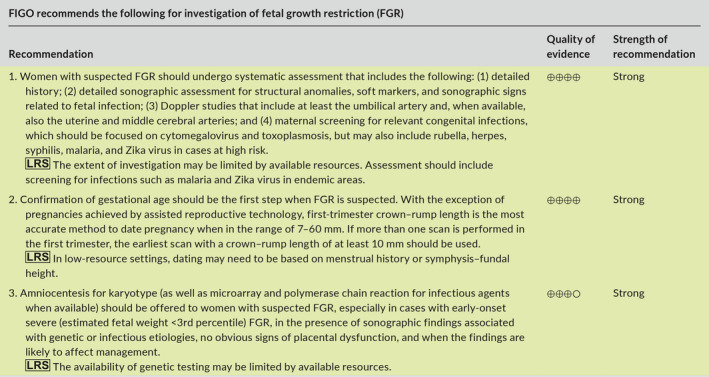

## 8 MANAGEMENT OF PREGNANCIES WITH FETAL GROWTH RESTRICTION

Management of pregnancies with FGR depends in part on the results of the investigation described in section 7. In cases of fetal abnormalities (genetic or infectious) the management (expectant versus pregnancy termination) should be individualized based on the nature of the disorder, the expected prognosis, gestational age, parental wishes, and local policies.

The most common underlying etiology for FGR is placental dysfunction. In early‐onset FGR (<32 weeks), increased resistance in umbilical artery Doppler is the primary rate‐limiting step to subsequent deterioration of cardiovascular and biophysical parameters.^286^, ^287^, ^288^, ^289^, ^290^, ^291^, ^292^ The primary management challenge arises from the risk of fetal deterioration and stillbirth in pregnancies undergoing surveillance versus the neonatal morbidity and mortality associated with preterm delivery.^68^, ^293^, ^294^, ^295^, ^296^, ^297^ In late‐onset FGR (≥32 weeks), cardiovascular deterioration in response to fetal hypoxia is predominantly confined to the cerebral circulation with little umbilical artery Doppler changes.^289^, ^291^, ^292^, ^298^ Pregnancies complicated by late‐onset FGR are major contributors to adverse perinatal outcome attributable to FGR because of misdiagnosis and challenges in detecting deterioration during fetal surveillance.^299^, ^300^

**FIGURE 5:**
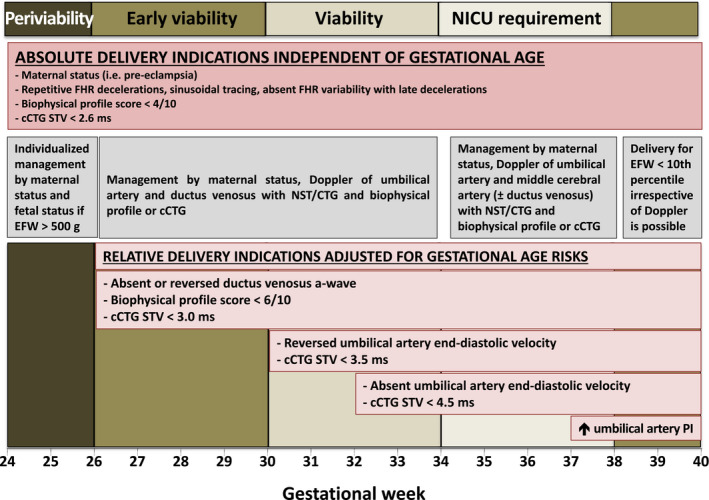
Delivery criteria for fetal growth restriction. Delivery criteria are based on monitoring with umbilical artery, ductus venosus, and middle cerebral artery Doppler at specified gestational ages with traditional nonstress testing or computerized CTG (cCTG) if available. Abbreviations: NICU, neonatal intensive care unit; FHR, fetal heart rate; CTG, cardiotocogram; STV, short‐term variation; ms, milliseconds; EFW, estimated fetal weight; PI, pulsatility index. [Colour figure can be viewed at wileyonlinelibrary.com]

There is no effective antenatal treatment for placental dysfunction and therefore once FGR has been identified, the principal management steps are institution of fetal surveillance and determination of appropriate thresholds for delivery. Perinatal outcome in early‐onset FGR is improved when pregnancies are managed in a high‐level fetal medicine and neonatology unit utilizing a uniform management protocol.^301^ Likewise, the optimal management setting for late‐onset FGR is a unit that has access and experience in interpretation of surveillance tests, together with an appropriate level neonatal unit. The recommendations for monitoring, timing and mode of delivery, and potential treatments for placenta‐mediated FGR are described below and summarized in Table 1 and Figure 5.

**TABLE 1:** Recommendations for monitoring, timing, and mode of delivery in cases with suspected fetal growth restriction.

Findings	Risk of stillbirth	Suggested monitoring^a^	Timing and mode of delivery^b^
SGA (EFW at 3rd–9th percentile, normal fluid and Doppler studies)	Low	Doppler (UA, MCA) every 1–2 weeks Growth every 2 weeks At ≥37 weeks consider BPP/NST 1–2 times per week^c^	37–39 weeks Mode of delivery: induction
Uncomplicated FGR at <3rd percentile (normal fluid and Doppler studies)	Low	Doppler (UA, MCA) 1–2 times per week Growth every 2 weeks At ≥37 weeks consider BPP/NST 1–2 times per week^c^	36–38 weeks Mode of delivery: induction
FGR with mild abnormalities: Early Doppler changes: UA PI >95th percentile, or MCA PI <5th percentile, or CPR <5th percentile, or UtA PI >95th percentile Oligohydramnios Suboptimal interval growth Suspected pre‐eclampsia	Low	Consider inpatient monitoring Consider steroids for fetal lung maturation BPP/NST 1–2 times per week Doppler (UA, MCA, DV) 1–2 times per week Growth every 2 weeks	34–37 weeks Mode of delivery: cesarean section or induction
FGR with umbilical artery AEDV/REDV	Overall risk of stillbirth^332^: AEDV: 6.8%, OR 3.6 [2.3–5.6] REDV: 19%, OR 7.3 [4.6–11.4] Risk of stillbirth with strict monitoring protocol with a safety net^343^: AEDV: 0%–1% REDV: 1%–2% Median time for deterioration: AEDV: 5 days REDV: 2 days	Inpatient monitoring Steroids for fetal lung maturation BPP/NST 1–2 times per day Doppler (UA, MCA, DV) every 1–2 days Growth every 2 weeks	AEDV: 32–34 weeks^d^ REDV: 30–32 weeks^d^ Mode of delivery: cesarean section
FGR with abnormal ductus venosus Doppler	Overall risk of stillbirth^332^: 20%, OR 11.6 (6.3–19.7) Risk of stillbirth with strict monitoring protocol with a safety net^343^: Elevated DV PIV: 2% Absent‐reverse a‐wave in DV: 4%	Inpatient monitoring Steroids for fetal lung maturation BPP/NST twice per day Daily Doppler	26–30 weeks^d^ Mode of delivery: cesarean delivery

Abbreviations: AEDV/REDV, absent or reversed diastolic velocity in the umbilical artery; BPP, biophysical profile; CPR, cerebroplacental ratio; DV, ductus venosus; FGR, fetal growth restriction; MCA, middle cerebral artery; NST, nonstress test; OR, odds ratio; PI, pulsatility index; PIV, pulsatility index for veins; SGA, small for gestational age; UA, umbilical artery; UtA, uterine artery.

^a^ Monitoring should be based on integration of multiple modalities (Doppler, BPP, NST).

^b^ Absolute indications for delivery at any gestational age and birth weight combination that are considered to be viable include: BPP or NST abnormalities or severe pre‐eclampsia with uncontrolled hypertension or end‐organ damage (section 8.2.3). In addition, timing of delivery should be individualized based on factors such as parental decision regarding threshold for intervention.

^c^ There is lack of evidence on the appropriate test to predict the risk of fetal deterioration and on the optimal monitoring strategy in cases of uncomplicated SGA fetuses, especially at term. Given this, there are differences in practice in various regions of the world regarding use of BPP/NST for fetal monitoring in this context, and some of the authors of these guidelines do not use BPP or NST for monitoring of fetuses with uncomplicated SGA as long as Doppler studies are normal. We suggest that the decision regarding use of BPP/NST should be based on local practices, the risk profile of the local population, and the available resources in each particular setting.

^d^ Timing should be individualized based on local neonatal outcomes. Before 26 weeks, careful and shared decision making with the parents and neonatology team is recommended.

### 8.1 Monitoring

The primary goal of fetal monitoring is prevention of stillbirth by detection of fetal deterioration that precedes irreversible compromise. To achieve this goal, monitoring tests need to be accurate in identifying fetal risks that favor delivery and, for pregnancies where delivery thresholds are not met, follow‐up monitoring needs to be frequent enough to provide a safety net against unanticipated deterioration or stillbirth. Fetal surveillance tests include maternal monitoring of fetal movements, cardiotocography, ultrasound evaluation of amniotic fluid volume and fetal activity, and Doppler ultrasound of the fetal arterial and venous circulations. With progressive compromise, abnormal fetal activity and fetal heart rate patterns are observed, independent of gestational age at diagnosis of FGR.^288^, ^292^, ^302^, ^303^, ^304^ In contrast, cardiovascular manifestations of fetal compromise are driven by placental blood flow resistance in the umbilical artery, and therefore differ significantly between early‐ and late‐onset FGR.^286^, ^289^, ^290^, ^291^, ^298^, ^299^ The surveillance tests that have been evaluated in the management of FGR pregnancies are described below.

#### 8.1.1 Fetal movement counting

Fetal activity is established from the first trimester onward and as gestational age advances becomes organized into coordinated behavioral states. Progressive fetal hypoxemia is accompanied by a reduction of fetal activity that can be perceived most accurately by the mother when she is lying down and paying focused attention to fetal movements.^305^ Decreased fetal movement is often defined as less than 10 movements in 2 hours during focused maternal counting.^306^ Although reports on whether quality improvement tools to promote awareness and management of reduced fetal movements can effectively decrease the risk of stillbirth have been conflicting, most of these interventions were focused on unselected populations rather than in pregnancies with suspected FGR.^307^, ^308^, ^309^ Given that fetal movement counting is a simple and inexpensive tool that may provide a safety net between scheduled outpatient monitoring visits, it seems reasonable to use movement counting as an adjunct to monitoring in FGR. The mother should be provided with clear behavioral instructions and confirmatory monitoring should be performed for patients presenting with decreased fetal movements.^305^, ^308^

#### 8.1.2 Fetal heart rate monitoring

Fetal heart rate monitoring is universally recommended to monitor pregnancies complicated with FGR.^134^, ^310^, ^311^, ^312^ Antepartum cardiotocography (CTG), also known as a nonstress test (NST), can be performed as a standalone evaluation or in conjunction with measurement of amniotic fluid volume (modified biophysical profile), or a five‐component biophysical profile (BBP).

Some heart rate characteristics reflect fetal oxygenation, gestational age, and maturational state of the nervous and cardiovascular systems. The normal heart rate baseline is between 110 and 160 beats per minute (bpm) and decreases with advancing gestation. Periodic accelerations of fetal heart rate (FHR) usually coincide with fetal movements, are observed from the early second trimester, and increase in magnitude and duration with advancing gestation. These are defined as increases in FHR over the baseline of at least 15 bpm and 15 seconds’ duration. Two or more of these accelerations define a “reactive” pattern. Recognizing that the frequency of reactivity increases from 50% at 24–28 weeks to 85% at 28–32 weeks of gestation, criteria of greater than or equal to 10 bpm amplitude and greater than 10 seconds’ duration are recommended at earlier gestational ages.^313^, ^314^, ^315^, ^316^ A “nonreactive” FHR pattern is one that does not display accelerations over an observation period of 40 minutes. In addition to reactivity, FHR patterns display “variability”—the average oscillations in the FHR signal, evaluated in bpm in 1‐minute windows. Reduced variability appears later than absent reactivity in the process of progressive fetal hypoxia. It reflects reduced sympathetic–parasympathetic activity, secondary to diminished brainstem oxygenation.

The FHR pattern reflects fetal oxygenation and acid‐base status at the time of evaluation but does not predict deterioration in FGR. In unselected pregnancies the rate of stillbirth in the week following a reactive CTG/NST is 1.9/1000 (negative predictive rate of 99.8%).^317^ Without any additional information, the empirically recommended minimum frequency is twice weekly CTG/NST. The frequency may be increased when evaluation of amniotic fluid or Doppler parameters indicate a more advanced degree of fetal compromise and delivery criteria have not yet been met. A nonreactive CTG/NST has low specificity for hypoxia and requires additional tests to determine fetal status and distinguish FHR pattern variations caused by fetal behavior, while reduced variability is a much stronger predictor of central nervous system hypoxia.

#### 8.1.3 Computerized fetal heart rate monitoring

Some professional societies recommend computerized fetal heart rate monitoring (cCTG) as the preferred modality to analyze CTG/NST tracings.^134^, ^308^ Inconsistency in visual assessment, particularly FHR variability, is a major contributor to interobserver variations in interpretation of CTG/NST tracings.^318^ cCTG evaluates FHR parameters such as baseline, accelerations, decelerations, and variability in an objective and quantifiable way. The Sonicaid cCTG system (Huntleigh Healthcare, Cardiff, UK) provides the parameter “short‐term variation” (STV) in milliseconds, while others quantify variability in a more traditional way in bpm.^319^ In contrast to visual FHR analysis, cCTG decreases observer variability and allows longitudinal numerical analysis of variability.^320^

FHR variability increases with gestational age; after 29 weeks of gestation, below 4.0 ms or below 3.0 ms meet criteria for reduced or very low STV, respectively.^321^ Before 29 weeks of gestation, STV below 3.5 ms is considered reduced, and below 2.6 ms is considered very low. STV below 3 ms has a 77% positive predictive value for fetal acidemia.^322^, ^323^

Similar to CTG/NST, cCTG does not predict fetal deterioration. In early‐onset FGR the daily risks for abnormal STV are 4%–5% but are unpredictable by additional monitoring tests. Accordingly, CTG/NST or cCTG monitoring needs to be performed more frequently than Doppler assessments. In patients receiving inpatient monitoring, a minimum frequency of daily cCTG/CTG/NST is recommended.^320^

#### 8.1.4 Ultrasound measurement of amniotic fluid volume

Professional societies do not recommend inclusion of isolated amniotic fluid volume assessment into management decisions for FGR. A decrease in amniotic fluid volume can occur as a result of fetal oliguria in response to progressive placental dysfunction and hypoxia, as well as rupture of membranes.^287^, ^288^, ^324^ Accordingly, additional evaluation is required to determine the significance of decreased amniotic fluid volume. Oligohydramnios can be defined as an ultrasound measured four‐quadrant amniotic fluid index below or equal to 5 cm, or a maximum vertical amniotic fluid pocket below or equal to 2 cm.^325^ Use of the latter reduces overdiagnosis of oligohydramnios and is preferred. Oligohydramnios is associated with an increased rate of intrapartum FHR abnormalities, need for cesarean section, and low 5‐minute Apgar scores, but not acidosis at birth.^326^

#### 8.1.5 Biophysical profile scoring

Biophysical profile (BPP) scoring is not universally recommended as the primary surveillance tool for FGR and is predominantly utilized in Canada and North America where the concept was first developed for fetal surveillance in the later part of the third trimester. The modified BPP refers to combined use of the CTG/NST as a short‐term indicator of fetal acid‐base balance and the maximum amniotic fluid pocket as an indicator of long‐term placental function.^327^ The five‐component BPP comprises fetal breathing movements, gross body movements, and tone, in addition to CTG/NST and maximum amniotic fluid pocket, and therefore includes four indicators of short‐term acid‐base balance.^301^

The modified BPP is considered abnormal when either the CTG/NST is nonreactive, or the maximum amniotic fluid pocket is below 2 cm. The most common reason for an abnormal modified BPP is a nonreactive CTG/NST, requiring additional ultrasound observation to complete a five‐component BPP and determine fetal acid‐base balance. The BPP is scored over a 30‐minute ultrasound observation period of the fetus. Fetal breathing movements are considered present if one or more episodes of 30 seconds of breathing or hiccups are observed. Fetal body movement is present when three or more discrete body or limb movements are observed. Fetal tone is present when one or more episodes of extension and flexion of the fetal extremities are observed. Each component of the BPP receives a score of 2 for its presence and 0 for its absence. Scores of 8–10, 6, and 4 or less are considered normal, equivocal, and abnormal, respectively.

In unselected pregnancies, the rate of stillbirth in the week following a normal modified or five‐component BPP is 0.8/1000 (negative predictive rate >99.9%). FGR fetuses show a sequential loss of heart rate reactivity, breathing movements, gross body movement, and tone with decrease in pH.^287^, ^288^, ^301^, ^302^ In FGR pregnancies, an abnormal BPP (score of 4 or less) is associated with an umbilical artery pH of less than 7.20, with sensitivity increasing to 100% at a score of 0/10.^301^, ^302^, ^319^

The BPP is a more accurate predictor of fetal acid‐base status at the time of testing than CTG/NST, with a similar accuracy as cCTG. Therefore, a five‐component BPP can be used to clarify fetal acid‐base status when a nonreactive CTG/NST is obtained. The frequency of BPP testing is guided by the same principles as timing of fetal heart rate testing.

#### 8.1.6 Umbilical artery Doppler

Umbilical artery Doppler is universally recommended for monitoring of FGR because it assesses the hemodynamic aspect of placental dysfunction.^134^, ^143^, ^308^, ^310^ It is estimated that approximately one‐third of the villous circulation needs to be damaged before a decrease in umbilical artery end‐diastolic velocity occurs. Absent or reversed umbilical artery end‐diastolic velocity corresponds to malperfusion of 50%–70% of the villous vascular tree.^328^ Because elevated villous blood flow resistance is predominantly associated with the placental pathology found in early‐onset FGR, umbilical artery Doppler does not reliably predict outcome in late‐onset FGR.^329^, ^330^, ^331^

The umbilical artery Doppler waveform can be quantified using the pulsatility index, or by visual classification of end‐diastolic velocity as absent (AEDV) or reversed (REDV). With increasing degrees of placental blood flow resistance, an abnormal umbilical artery waveform is defined as either having an elevated pulsatility index, AEDV, or REDV. The degree of placental blood flow resistance elevation is the primary factor determining the rate of clinical progression and the associated risk for fetal deterioration and stillbirth in early‐onset‐FGR.^286^, ^289^, ^291^, ^292^ When the umbilical artery pulsatility index is elevated but end‐diastolic forward flow is still present, the median time interval to additional surveillance abnormalities is 2 weeks. Once AEDV occurs, cardiovascular deterioration advances after a median of 5 days and the weighted odds ratio for stillbirth is 3.6 (2.3–5.6).^286^, ^291^, ^332^ When REDV occurs, the median interval for further fetal deterioration is 2 days and the weighted odds ratio for stillbirth is 7.3 (4.6–11.4).^291^, ^331^

In patients with normal umbilical artery Doppler, the recommended frequency to repeat Doppler monitoring ranges from weekly to every other week. However, when AEDV develops, Doppler surveillance is recommended at minimum twice weekly, and for REDV at least three times weekly unless delivery criteria have been met.

#### 8.1.7 Cerebral artery Doppler

The majority of professional societies now recommend middle cerebral artery Doppler for monitoring in late‐onset FGR. Concurrent measurement of the umbilical artery and middle cerebral artery pulsatility index allows calculation of the cerebroplacental Doppler ratio. Both the cerebroplacental ratio and middle cerebral artery pulsatility index decrease as a hemodynamic response to fetal hypoxemia and therefore reflect placental dysfunction, even in those pregnancies where the villous blood flow resistance is not elevated enough to produce an abnormal umbilical artery pulsatility index. Approximately 20% of term SGA fetuses with normal umbilical artery Doppler have a decreased middle cerebral artery pulsatility index, which is associated with a higher rate of cesarean section for intrapartum distress, poor neonatal transition, and adverse developmental outcome.^333^, ^334^, ^335^ The cerebroplacental Doppler ratio is more closely related to fetal hypoxia than its individual components,^336^ but has a similar predictive accuracy for perinatal death, fetal distress, or poor neonatal transition as the umbilical artery pulsatility index.^337^

Cardiovascular deterioration in late‐onset FGR is characterized by abnormal cerebral artery Doppler. Therefore, an important role of middle cerebral artery Doppler is to provide an estimate of perinatal risk in patients with normal umbilical artery Doppler.^292^, ^331^ Because of the higher risk for adverse outcome within 1 week of a decrease in middle cerebral artery pulsatility index, it is recommended to utilize at least twice weekly surveillance in this setting.

#### 8.1.8 Ductus venosus Doppler

The few professional societies that recommend ductus venosus Doppler evaluation specify that it should be performed in specialized centers that have expertise in the comprehensive perinatal management of early‐onset FGR.^312^ The relative forward flow in atrial systole in the ductus venosus decreases with worsening placental function or reduced fetal cardiac function, leading to an increase in the pulsatility index for veins, absent, or reversal of the a‐wave.^286^, ^288^, ^291^, ^292^, ^338^

Abnormal ductus venosus Doppler is primarily observed in early‐onset FGR and can provide an estimate of fetal acid‐base balance and the risk of stillbirth. The odds ratio of absent or reversed atrial systolic velocity for an umbilical artery pH less than 7.20 at birth is 4.4 (1.2–17.2).^339^, ^340^ The weighted odds ratio of absent or reversed ductus venosus atrial systolic velocity for fetal death is 11.6 (6.3–19.7).^331^

Abnormal ductus venosus Doppler also predicts fetal decompensation to an abnormal BPP, reduced variability on cCTG, or stillbirth. In fetuses with elevated ductus venosus pulsatility index for veins but forward flow during atrial systole, the median interval to progressive venous Doppler deterioration can be as short as 2 days.^291^ In patients that do not yet meet delivery criteria, ductus venosus Doppler is recommended at minimum twice weekly in patients with AEDV and three times weekly when REDV is observed.^286^, ^291^, ^292^, ^341^ When ductus venosus Doppler indices increase as a new finding, the frequency of monitoring needs to be increased further.

#### 8.1.9 Surveillance strategy

Monitoring in FGR pregnancies is intended to prevent fetal compromise or stillbirth, and the choice of tests and their timing is heavily influenced by gestational age. A robust plan is essential, since expectant management with ongoing monitoring, particularly in the setting of early‐onset FGR, can result in a three to five‐fold increased stillbirth rate when compared with immediate delivery, depending on the degree of cardiovascular compromise that is tolerated before triggering delivery.^294^, ^342^, ^343^ The optimal monitoring frequency in FGR has not been determined due to the varying circumstances of gestational age and severity of FGR. A combination of surveillance modalities is needed to accurately determine fetal acid‐base status at the time of testing, as well as allowing anticipation of future deterioration.^289^, ^290^, ^291^, ^292^, ^298^, ^344^ The accurate prediction of fetal acid‐base status is required to prevent unnecessary intervention and nonindicated delivery. The anticipation of deterioration informs subsequent monitoring intervals that provide a safety net against unanticipated fetal acidosis and asphyxia. The combination of biophysical (CTG/NST, cCTG, BPP) and cardiovascular parameters (umbilical artery, middle cerebral artery, and ductus venosus Doppler) is considered a robust approach for FGR surveillance. Among these modalities, the combination of CTG/NST and umbilical artery Doppler is universally recommended.

There is good evidence that umbilical artery Doppler offers sufficient information to determine monitoring frequency in early‐onset FGR. Although middle cerebral artery Doppler could provide additional information in those late‐onset FGR pregnancies with normal umbilical artery Doppler, this practice has not been evaluated.^292^, ^328^, ^329^ Based on observational data in term FGR with normal umbilical artery Doppler, the approximate interval to stillbirth in patients with abnormal middle cerebral artery Doppler is 4 days, suggesting the need for twice weekly CTG/NST monitoring. In the absence of further evidence on the clinical benefit of middle cerebral artery Doppler, twice weekly CTG/NST monitoring in FGR after 32 weeks of gestation in patients with normal umbilical artery Doppler provides the same safety net (Table 1).

When umbilical artery pulsatility index is elevated, weekly Doppler is suggested, and when there is AEDV or REDV, more frequent assessment is recommended (Table 1). In early‐onset FGR with AEDV or REDV, the risk of stillbirth increases when the ductus venosus Doppler or the CTG/NST patterns become abnormal.^292^, ^319^, ^340^ However, there is currently no evidence that adjusting the timing of monitoring based on ductus venosus Doppler improves outcome. In patients with AEDV the stillbirth rate is 0%–1% when at least once daily CTG/NST, cCTG, or BPP are performed with predefined delivery criteria.^341^, ^342^ When monitoring is continued to allow for an increase in the ductus venosus pulsatility index for veins, the stillbirth rate is 2%, and 11% of deliveries occur for abnormal STV, 19% for an abnormal BPP, and 22% for FHR decelerations.^304^, ^342^ When monitoring is continued in anticipation of reversal of the ductus venosus a‐wave velocity, the stillbirth rate is 4%, and 20% of deliveries occur for abnormal STV, 29% for an abnormal BPP, and 31% for FHR decelerations (Table 1).^342^, ^345^ This indicates that with ongoing monitoring the risk of FHR abnormalities or an abnormal BPP requiring delivery cannot be predicted by the ductus venosus Doppler.^319^, ^346^ Based on the regional pattern of practice, this indicates that in patients who are admitted for AEDV, the minimum frequency of CTG/NST or BPP should be daily and more frequent with REDV (Table 1).^319^

### 8.2 Timing of delivery

The timing of delivery in FGR is determined by gestational age, severity of FGR, findings of fetal monitoring tests, and maternal factors such as pre‐eclampsia (Table 1 and Figure 5). Delivery indications can be considered as absolute if they are independent of gestational age, and relative if the threshold to deliver based on the surveillance findings varies across gestational age.

#### 8.2.1 Gestational age‐related risks in fetal growth restriction

With advancing gestational age there are several important changes in the relative risks of delivery versus ongoing surveillance that define the delivery thresholds.

From 24–28 weeks of gestation each day of pregnancy prolongation results in an estimated 2% decrease in neonatal death, as well as major neonatal complications including bronchopulmonary dysplasia, high‐grade intraventricular hemorrhage, and surgical necrotizing enterocolitis. The impact of prematurity, neonatal weight below 500 g, challenging resuscitation, and decreased tolerance for low Apgar scores results in average neonatal survival rates below 50% and intact survival below 50% until 26 weeks.^68^, ^298^, ^345^, ^346^

Between 28 and 30 weeks of gestation the daily increment in survival is approximately 0.7%. After 30 weeks, neonatal survival rates exceed 90%,^68^, ^296^, ^297^ and there is a significant decrease in major neonatal complications from approximately 35% at 30 weeks to less than 10% at 34 weeks, as well as a decrease in the risk of neurodevelopmental delay for neonates delivered after this time. FGR infants delivered prior to 30 weeks have a three‐fold higher rate of developmental abnormalities and an up to eight‐fold increased rate of cerebral palsy.^10^, ^295^

From 34–38 weeks of gestation neonates are more likely to require admission to the intensive care nursery but have reduced risks of major neonatal complications.^347^, ^348^ In SGA fetuses that remain undelivered after 38 weeks, the risk of stillbirth doubles every week and reaches 60/10 000 for pregnancies that continue beyond the due date.^349^, ^350^

#### 8.2.2 Gestational age‐related management strategy

The balance between fetal and neonatal risks defines the predominant management strategy at different gestational epochs. Accordingly, the goal of management shifts from gaining fetal viability at 26 weeks to a graded improvement in survival, neonatal morbidity, and neurodevelopment by delaying delivery until 34–36 weeks. The increase in stillbirth rate in undelivered fetuses increasingly favors delivery from 36 weeks onward.

Timing of delivery in FGR has been evaluated in three randomized trials. The growth restriction intervention trial (GRIT) randomized pregnancies that had abnormal fetal biometry and umbilical artery Doppler studies performed as part of clinical management into immediate delivery after completion of a course of steroids versus delivery when the managing physician was no longer comfortable with conservative management.^294^, ^295^ The monitoring protocol and delivery criteria were not specified. The trial demonstrated that, in the absence of specific criteria, either management approach resulted in the same perinatal outcome. Delaying delivery increased the risk of stillbirth, while earlier delivery resulted in a higher degree of prematurity‐related complications that either led to neonatal death or an increased risk of developmental delay.

The disproportionate intrauterine growth intervention trial at term (DIGITAT) randomized SGA fetuses by several biometry criteria, independent of the umbilical artery Doppler pattern, to induction or expectant monitoring between 36 and 41 weeks of gestation.^347^ The study demonstrated that, while elective induction did not affect neonatal or obstetric outcomes, deliveries prior to 38 weeks resulted in a higher rate of admissions to the neonatal intensive care unit.

These trials demonstrate that the relative risk for neonatal complications requires definitive delivery indications until 38 weeks of gestation. After that time, delivery for indication of FGR is likely to prevent stillbirth in ongoing pregnancies. The continuous decrease in neonatal risks requires that delivery indications at early gestational ages occur at a higher threshold for fetal risks than after 30–32 weeks.

#### 8.2.3 Absolute delivery criteria for fetal growth restriction (independent of gestational age)

Absolute delivery criteria are findings associated with important health risks to the mother or fetus, and therefore require delivery without consideration of gestational age (Figure 5).

The fetal biophysical variables are strongly influenced by oxygen tension in the regulatory centers. A 30‐minute BBP score of 0 or 2, or a 60‐minute score of 4 indicates a prelabor fetal pH of less than 7.20 and requires delivery to prevent fetal demise.

Repetitive FHR decelerations, a sinusoidal heart rate, absent variability with recurrent late decelerations, or bradycardia predict fetal acidemia and poor perinatal outcome and require delivery if the causative stimulus cannot be removed. When cCTG is used, a short‐term variation below 2.6 ms is below the 5th percentile irrespective of gestational age and requires delivery for its strong association with fetal acidemia.

Maternal pre‐eclampsia with severe features complicates up to 30% of FGR pregnancies, with a higher proportion in early‐onset FGR. In the absence of effective treatment other than delivery, pre‐eclampsia with uncontrolled severe hypertension, HELLP syndrome (hemolysis, elevated liver enzyme levels, and low platelets), or other evidence of end‐organ damage (e.g. oliguria or acute renal injury other than proteinuria, pulmonary edema, or eclampsia) requires delivery (Figure 5).

#### 8.2.4 Relative delivery criteria for fetal growth restriction (adjusted for gestational age)

The trial of umbilical and fetal flow in Europe (TRUFFLE) evaluated two monitoring strategies and specific delivery criteria in early‐onset FGR, with survival without neurodevelopmental impairment at 2 years of age as the primary outcome.^343^ Monitoring with cCTG and umbilical artery Doppler was universal in all patients, while ductus venosus Doppler was only added in two study arms. Patients were randomized to one of three specific delivery criteria: (1) abnormal cCTG STV; (2) mild ductus venosus abnormalities; and (3) severe ductus venosus abnormalities with absence or reversal of the a‐wave. Because patients with ductus venosus Doppler monitoring also had FHR monitoring, safety net delivery criteria based on cCTG were also applied in these groups. These included STV below 2.6 ms irrespective of gestational age and below 3.0 ms from 29 weeks onward. In addition, umbilical artery Doppler findings were utilized as relative delivery criteria from 30 weeks onward for REDV and 32 weeks onward for AEDV. The choice of these thresholds is supported by a recent meta‐analysis that found that in undelivered FGR pregnancies, umbilical artery REDV has a 19% stillbirth rate, which exceeds mortality for neonates delivered from 30 weeks onward, while AEDV carries a 6.8% stillbirth risk, which favors delivery due to lower neonatal mortality from 32 weeks onward (Table 1 and Figure 1).^332^

The TRUFFLE study demonstrated that a predefined management strategy produces better outcomes than expected in all FGR pregnancies.^343^ The primary endpoint was less frequently observed in patients randomized to deliver for late ductus venosus abnormality. Overall, cCTG was the most frequent trigger for delivery. In the three arms, delivery was based on abnormal STV in 11%–51% of participants, and visually apparent FHR decelerations led to delivery in 22%–31% of participants. While the strategy of awaiting absent or reversed ductus venosus a‐wave to determine delivery produced the better study outcome, it is noteworthy that the stillbirth rate increased four‐fold compared with patients who were monitored with cCTG and umbilical artery Doppler. In addition, an absent ductus venosus a‐wave only triggered delivery in 10% of participants in this study arm. The frequency of delivery decisions based on FHR abnormalities emphasizes the importance of concurrently monitoring growth‐restricted fetuses with more than one modality.

Because cCTG is not universally available, most healthcare providers need to rely on traditional CTG/NST monitoring. While BPP has not been studied in randomized intervention trials in FGR, it is an established monitoring tool to verify fetal status in patients with a nonreactive tracing. In FGR, an abnormal BPP predicts abnormal arterial pH with a similar accuracy to cCTG and is an independent delivery trigger at a comparable frequency to cCTG.^304^, ^319^ Therefore, it is recommended that in FGR fetuses with nonreassuring CTG/NST not yet meeting the criteria for delivery, a BPP is completed to establish fetal status. If the expertise is not available to perform a BPP, prolongation of the CTG/NST or increase in testing frequency may be required to determine if delivery is necessary.

Optimal delivery criteria for FGR presenting after 32 weeks of gestation have not been evaluated in a randomized trial and are based on expert consensus. Table 1 and Figure 5 summarize the management approaches and recommendations for delivery. When local neonatal outcomes are consistently more favorable for FGR neonates, relative delivery indications may be applied at earlier gestational ages than indicated. For example, improved neonatal survival may justify delivery for REDV from 30 weeks onward.

### 8.3 Mode of delivery and intrapartum considerations

FGR in itself is not an indication for cesarean section. However, primary cesarean section may be considered in selected cases of severe FGR where the likelihood of successful vaginal delivery is low.

Fetuses with placenta‐mediated FGR are less likely to tolerate the stress associated with labor and are at increased risk of requiring intrapartum urgent cesarean section for nonreassuring FHR tracing. Therefore, in certain cases of FGR, a trial of labor is highly unlikely to be successful and might be associated with fetal risks to the extent that a primary cesarean section should be preferred. This depends on multiple factors including gestational age, severity of FGR, Doppler changes, associated pre‐eclampsia, parity, cervical Bishop score, and patient preference (Table 1).

In cases of early‐onset FGR the main goal is to prolong pregnancy and maximize fetal maturation by means of expectant management under close monitoring until there is evidence of late Doppler changes in the umbilical artery (AEDV or REDV), ductus venosus alterations, or FHR abnormalities. Therefore, at the point when delivery is indicated in cases of severe early‐onset FGR, the fetus might already be experiencing some degree of hypoxia or acidosis,^291^ in which case the likelihood of the fetus tolerating labor is low and the rate of cesarean section has been reported to be greater than 80%.^351^ In addition, labor induction in general is less likely to be successful during the preterm period.^352^, ^353^ For these reasons, primary cesarean section is usually the preferred option when delivery is indicated in cases of severe early‐onset FGR. ^354^

In contrast, late‐onset FGR is usually less severe and fetal hypoxia or acidosis are less likely to be present at the time when delivery is indicated. Indeed, in the DIGITAT trial the rate of vaginal delivery was greater than 80% in pregnancies induced for SGA with normal umbilical artery Doppler after 36 weeks of gestation.^347^ This observation suggests that most term SGA fetuses with normal umbilical artery Doppler can tolerate labor and that the presence of late‐onset FGR in the absence of additional factors does not preclude induction of labor. Several studies have tried to individualize the decision regarding mode of delivery through the development of models for the prediction of urgent cesarean section in women with late‐onset SGA undergoing labor induction. The factors that were most predictive of urgent cesarean section were gestational age, severity of SGA (EFW <3rd percentile), cerebral Doppler (middle cerebral artery and cerebroplacental ratio), and Bishop score.^355^, ^356^ For example, in a large cohort study of 509 women undergoing labor induction for late‐onset SGA, the predictive model had a positive predictive value of 36% and a negative predictive value of 89% for urgent cesarean section for nonreassuring fetal state.^355^ Thus, although this information can be helpful for patient counselling regarding mode of delivery and may reassure women with none of these risk factors of the high likelihood of a successful trial of labor (nearly 90%), the positive predictive value of these models (i.e. a risk of cesarean section in the range of 30%–40%) is not high enough to preclude a trial of labor even when these risk factors are present.

The optimal approach for cervical ripening in women undergoing induction for FGR remains unclear. In a recent meta‐analysis of 12 trials on cervical ripening in pregnancies complicated by SGA or FGR, the authors concluded that mechanical methods (such as balloon catheters) seem to be associated with a lower risk of cesarean section and intrapartum complications compared with alternatives such as dinoprostone.^357^ Given these data, it seems reasonable to prefer balloon catheter over prostaglandin preparations, when possible, for cervical ripening in pregnancies with suspected FGR. If prostaglandin agents are used, a reversible method (e.g. dinoprostone vaginal insert) should be preferred.

During labor, continuous FHR monitoring is recommended. Delivery should take place at an institution with the appropriate level of neonatal care for the gestational age and the anticipated management needs of the neonate.

It is recommended that the placenta is sent for histopathological evaluation after delivery. Ideally this should be done in accordance with the Amsterdam workshop consensus statement. ^358^ High‐quality evaluation of the placental pathology is not only likely to increase the precision of the diagnosis but also provides information on the risks of recurrence.^18^, ^359^, ^360^

### 8.4 Medical interventions

#### 8.4.1 Antenatal corticosteroids

The efficacy of antenatal corticosteroids in cases of FGR has been questioned, based on reports of elevated endogenous cortisol levels in this population when compared with normally grown fetuses.^361^, ^362^, ^363^, ^364^ In addition, the unique cardiovascular, hormonal, and metabolic changes characteristic of growth‐restricted fetuses ^276^, ^365^, ^366^, ^367^, ^368^, ^369^ have raised concerns that exposure to exogenous steroids may produce potentially harmful cardiovascular and metabolic effects in these already compromised fetuses. Indeed, exposure to corticosteroids has been shown to result in Doppler changes in growth‐restricted fetuses such as transient increase in diastolic flow in the umbilical artery ^370^, ^371^, ^372^, ^373^ and the middle cerebral artery,^374^, ^375^, ^376^ which have been attributed to peripheral vasodilatation or an increase in cardiac output and circulatory stress.^376^, ^377^ Despite this, recent data support the efficacy and safety of antenatal corticosteroids in the subgroup of SGA fetuses,^378^, ^379^ which should be administered when delivery is anticipated, ideally within 1–7 days before birth.^380^ When administered in cases of severe FGR with late Doppler changes, an inpatient setting is advised where the fetus can be closely monitored. Finally, it is important to recognize that the “improvement” in umbilical artery Doppler that is often seen following administration of antenatal corticosteroids is transient, and is thought to be the result of vasodilation of the fetoplacental arterial tree and increased fetal cardiac output rather than a true decrease in placental resistance.^381^ Therefore, these transient changes should not be interpreted as an improvement in fetal status and should not affect the management plan. Of note, the absence of any change in end‐diastolic flow in response to antenatal corticosteroids is a concern and predicts subsequent fetal deterioration.^372^

#### 8.4.2 Magnesium sulfate for neuroprotection

Administration of magnesium sulfate to women at risk of preterm birth has been shown to have a neuroprotective role, with a decrease in the risk of perinatal mortality, cerebral palsy, and gross motor dysfunction.^382^, ^383^ Possible mechanisms thought to be involved in the beneficial effects of magnesium sulfate include reducing intracellular calcium levels, stabilizing blood pressure, normalizing cerebral blood flow, blocking the effects of excitatory neurotransmitters such as glutamate, and antioxidant and anti‐inflammatory effects.^384^, ^385^ However, the optimal protocol for the administration of magnesium sulfate for the purpose of neuroprotection remains unclear and available protocols vary with regard to the timing of administration, upper gestational age limit, dose, duration, and need for repeat doses.^386^, ^387^, ^388^, ^389^

The observation that term FGR infants have higher cord blood magnesium levels compared with normally grown infants raises the theoretical concern that maternal administration of magnesium sulfate in cases of FGR might result in toxic magnesium levels in the fetus.^390^, ^391^ However, there are currently no data on the efficacy and safety of magnesium sulfate in FGR fetuses that can support or refute these theoretical concerns. Therefore, there is currently no evidence in favor or against recommending administration of magnesium sulfate for neuroprotection in women at risk of preterm birth with suspected FGR.^379^ We believe that, at the current time, it is reasonable to extrapolate the efficacy of magnesium sulfate to specific subgroups of pregnancies, including those complicated by FGR, especially given that FGR is an independent risk factor for cerebral palsy.

#### 8.4.3 Treatments under investigation

Several novel therapies aiming to improve poor placentation and uterine blood flow are being explored, some of which are described below. However, there are currently no proven treatments for FGR, and any of the therapies currently under investigation should be evaluated only in an appropriately regulated research setting.^392^

Phosphodiesterase type‐5 inhibitors, such as sildenafil citrate, potentiate nitric oxide availability, lead to vasodilatation,,^393^, ^394^ and can improve umbilical artery and middle cerebral artery Doppler.^395^ However, in the recently published STRIDER trial, which randomized 135 women with early‐onset FGR to 25 mg sildenafil three times daily or placebo, sildenafil did not prolong pregnancy or improve pregnancy outcomes.^396^ More recently, a similar randomized trial was halted prematurely due to lack of benefit along with concerns that sildenafil may cause neonatal pulmonary hypertension.^397^

Another approach is to target the uteroplacental circulation with maternal vascular endothelial growth factor gene therapy, thereby improving local vasodilatation and angiogenesis.^392^ Clinically, vector delivery into the uterine arteries can be achieved with intervention radiology. This approach is currently being investigated in the ongoing EVERREST trial.^398^ Protein pump inhibitors have been shown in vitro to decrease sFlt‐1 and soluble endoglin and improve markers of endothelial dysfunction. However, in a recent randomized trial involving 120 women with preterm pre‐eclampsia, esomeprazole did not improve pregnancy outcomes.^399^ Pravastatin has been shown to have anti‐inflammatory, antioxidant, and proangiogenic properties.^400^, ^401^ However, in a recently published randomized trial of 94 women with early‐onset pre‐eclampsia, the administration of 40 mg pravastatin daily did not lower maternal sFlt‐1 levels or prolong pregnancy when compared with placebo.^402^ Other novel potential therapies include nanoparticles and microRNAs that deliver drugs locally to the uterine arterial endothelium or trophoblasts, to improve uterine blood flow and placental function.

### 8.5 Recommendations

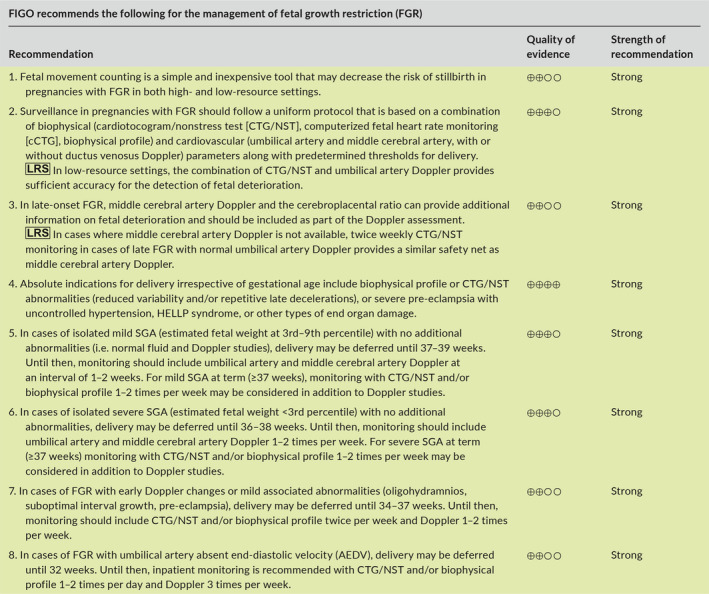

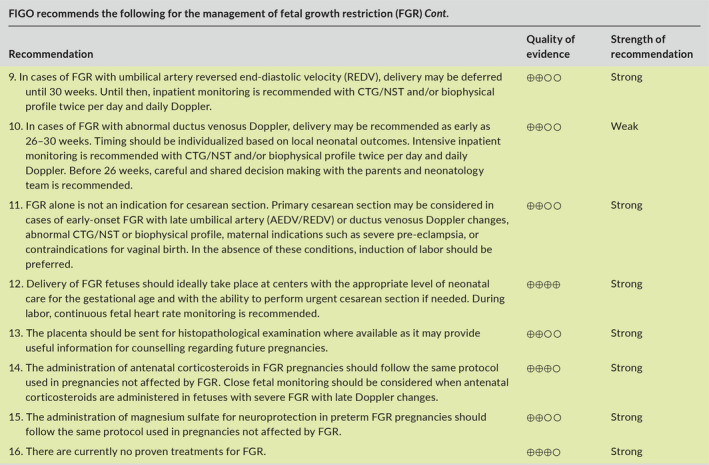

## 9 POSTPARTUM ASSESSMENT AND COUNSELLING FOR FUTURE PREGNANCIES

### 9.1 Infant follow‐up

Growth‐restricted infants are at increased risk of both immediate and long‐term complications, and therefore require closer follow‐up than normally grown infants in the first years of life.

Growth‐restricted infants have lower survival rates compared with those appropriate for gestational age.^403^ Although this may be attributed in part to prematurity that is often associated with FGR, birth weight has been shown to be an independent prognostic factor for neonatal mortality, irrespective of gestational age.^404^ In a population‐based cohort study, the mortality rate of term FGR neonates was approximately five‐fold higher compared with appropriate for gestational age neonates (0.3% vs 0.06%).^405^

FGR can affect postnatal growth. In cases of mild FGR, infants tend to achieve normal height during the first year of life.^406^ In cases affected by severe FGR, however, height in the late teens is lower than those born appropriate for gestational age (169.9 ± 1.5 vs 175.4 ± 0.8 cm; *p* < 0.0001 for boys; and 159.4 ± 1.3 vs 163.1 ± 0.8 cm; *p* < 0.0005 for girls).^407^

FGR infants are also at increased risk of adverse long‐term neurodevelopmental outcomes. A systematic review of this topic found that FGR infants are at higher risk of poor neurodevelopmental outcomes measured up to 3 years of age; however, high levels of heterogeneity in primary outcomes were reported in the studies included in the review.^12^ Of note, adverse neurodevelopmental outcomes may at least partly be related to coexisting increased prematurity rates.^408^

In line with the developmental origins of health and disease hypothesis, FGR has been associated, in both animal and human studies, with an increased risk of future noncommunicable diseases including obesity, diabetes, hypertension, and cardiovascular disease.^409^, ^410^, ^411^ The risk is especially high in those infants who experience rapid catch‐up growth in the first few years of life.^412^, ^413^ The mechanisms underlying these associations are not entirely clear. However, fetal programming by means of epigenetic changes as well as direct organ damage are thought to play a role.^414^ Ongoing studies are investigating the optimal follow‐up and prevention strategies to decrease the risk of these complications.^415^, ^416^

### 9.2 Maternal follow‐up

It is well established that women with a history of pregnancy complicated by FGR or other placenta‐mediated complications such as pre‐eclampsia are at an increased risk of future cardiovascular disease, especially in the presence of early‐onset disease. In a population‐based study that included more than 100 000 pregnancies and provided maternal follow‐up for 15–19 years, delivery of a low birth weight infant was associated with increased maternal risk of ischemic heart disease or death (aHR 1.9; 95% CI, 1.5–2.4).^417^ Moreover, a combination of FGR, pre‐eclampsia, and preterm delivery amplified the risk of disease seven‐fold. For a detailed review of the evidence supporting these associations, their underlying mechanisms, and recommendations on maternal follow‐up and prevention strategies please refer to the recently published FIGO postpregnancy initiative on long‐term maternal implications of pregnancy complications and follow‐up considerations.^4^

### 9.3 Counselling regarding future pregnancies

The most frequent and relevant question that care providers are being asked by couples whose prior pregnancy was complicated by FGR relates to the likelihood of a similar complication in subsequent pregnancies. The answer to this question is often difficult and depends on several factors, namely the underlying etiology, severity and timing of onset, and the presence or absence of modifiable risk factors (e.g. maternal medical conditions or smoking). In cases of placenta‐mediated FGR, the results of the placental histopathological examination may provide valuable information that can assist care providers in counselling patients regarding the risk of recurrence, role of further investigation, and potential preventive interventions in subsequent pregnancies.

#### 9.3.1 Risk of recurrence based on severity and onset

Most of the data on the risk of recurrence of placenta‐mediated complications come from studies evaluating hypertensive complications of pregnancy. In a recent individual patient data meta‐analysis of 22 studies, the overall risk of recurrence of hypertensive complications was 21%, and was higher in women who experienced early‐onset hypertensive complications.^418^ Data on the recurrence of FGR are limited.^419^, ^420^, ^421^, ^422^ In a population‐based study, the overall recurrence rate of FGR in women who gave birth to an infant with a birth weight below the 10th percentile was 24%, compared with 6% in women without a history of FGR (OR 3.9; 95% CI, 3.7–4.0). The risk of recurrence was related to the severity of FGR, and was nearly six‐fold when the infant birth weight was below the 5th percentile (OR 5.7; 95% CI, 5.4–6.0).^423^ Thus, couples with FGR in the first pregnancy can be reassured that the overall chance of recurrence in subsequent pregnancies is less than 25%. However, interpretation of the data is limited by the lack of distinction between constitutionally SGA infants and infants who were truly growth restricted, as much of the association described in that study may be driven by the recurrence of constitutional SGA. Therefore, counselling regarding the risk of recurrence should be further refined based on the risk factors of the individual patient, severity of FGR as reflected by timing of onset and Doppler findings, the co‐presence of pre‐eclampsia, and placental histopathological findings.

#### 9.3.2 Risk of recurrence based on placental histopathology

The results of the placental histopathological examination are important for two main reasons. First, they may assist care providers in counselling couples regarding the most likely etiology of FGR, especially when the clinical presentation and Doppler findings were inconclusive. Second, placental findings may provide valuable information regarding the risk of recurrence, as certain types of placental pathologies are associated with a relatively high recurrence rate. The main types of placental pathologies, the clinical phenotypes associated with these pathologies, and their estimated risks of recurrence are summarized in Table 2.^424^, ^425^, ^426^

**TABLE 2:** Phenotypes and risk of recurrence associated with specific types of placental pathologies.

Placental pathology	Incidence	Common placental findings	Pathophysiology	Phenotype	Risk of recurrence	Recommendations for investigation and prevention in next pregnancy	Refs
Maternal vascular malperfusion (MVM)	Common	Decidual arteriopathy, agglutinated villi, increased syncytial knots, intervillous fibrin deposition, villous infarcts	Placental malperfusion due to shallow trophoblast invasion and failure of remodeling of spiral arteries	Early‐ or late‐onset FGR, pre‐eclampsia, placental abruption	10%–25%	Screening for antiphospholipid antibodies may be considered in selected cases of severe early‐onset FGR, when placental examination shows features of severe MVM such as especially central or multiple areas of villous infarction Consider aspirin in subsequent pregnancy, especially if associated with pre‐eclampsia	173, 425
Fetal vascular malperfusion (FVM)	Relatively common	Avascular villi, chorionic plate or stem villous thrombi, obstructive lesions of umbilical cord	Most common cause: chronic/intermittent cord obstruction due to cord compression, entanglement, or hypercoiling. Possible association with hereditary thrombophilia	FGR, fetal CNS injury, stillbirth	Low	Consider screening of the infant or the mother for hereditary thrombophilia	454, 455, 456
Chronic inflammation							
Villitis of unknown etiology (VUE)	Relatively common (5%–10% of term placentas)	Chronic T‐cell mediated inflammation of villous stroma	Maternal graft versus host response to fetal antigens in the placenta	Late‐onset FGR, abnormal neurodevelopmental outcome, stillbirth	10%–50%		457, 458, 459, 460, 461, 462, 463, 464
Chronic histiocytic intervillositis	Rare	Maternal histiocytic infiltrate in the intervillous space		Recurrent miscarriages, recurrent severe early‐onset FGR, stillbirth	70%–100%	Suggested interventions include prednisone, hydroxychloroquine, aspirin, low‐molecular‐weight heparin Associated with increased levels of serum alpha‐fetoprotein and alkaline phosphatase	461, 463, 465, 466, 467, 468, 469
Massive perivillous fibrinoid deposition (maternal floor infarction)	Rare	Large amounts of fibrinoid matrix surrounding villi	Unclear	Recurrent miscarriages, recurrent severe early‐onset FGR, stillbirth	40%–60%	Consider screening for antiphospholipid antibodies, hereditary thrombophilia Anecdotal reports of treatment with aspirin, heparin, and IVIG	426, 463, 470

Abbreviations: CNS, central nervous system; FGR, fetal growth restriction; IVIG, intravenous immunoglobulins.

#### 9.3.3 Role of thrombophilia screening

Whether women who experienced placenta‐mediated pregnancy complications should be screened for antiphospholipid syndrome is a matter of debate. Although the consensus criteria for antiphospholipid syndrome include premature birth before 34 weeks for severe pre‐eclampsia or features consistent with placental insufficiency including birth weight below the 10th percentile,^427^ the association of antiphospholipid (aPL) antibodies with these conditions is relatively weak and conflicting, especially for FGR.^428^, ^429^, ^430^ In addition, although some care providers recommend treatment with LMWH during pregnancy to women with aPL syndrome and previous preterm birth for placenta‐mediated complications, this practice is mostly extrapolated from women with aPL syndrome and recurrent pregnancy loss, where there is some evidence in favor of LMWH.^431^, ^432^, ^433^ However, the only trial on LMWH in women with aPL syndrome and prior placenta‐related complications (FRUIT trial) found no evidence that LMWH improves outcomes in these cases.^434^ Given the above, there is insufficient evidence to justify routine screening for aPL antibodies in women with prior FGR.^435^ However, screening for aPL antibodies is recommended in women with a history of thromboembolism or recurrent pregnancy loss (or ≥1 late fetal loss), and may be considered in selected cases of women with a history of severe FGR associated with severe early‐onset pre‐eclampsia, when placental examination shows features of severe maternal vascular malperfusion, especially central or multiple areas of villous infarction that are due to multiple spiral artery thromboses.

Management of women already diagnosed with antiphospholipid syndrome based on a history of placenta‐mediated complications is also under debate. Based on the evidence from the FRUIT trial described above, some only recommend treatment with aspirin in this setting,^436^ while others recommend either surveillance or LMWH during the antepartum and postpartum periods.^437^ Based on available evidence we only recommend treatment with aspirin, and suggest that LMWH be considered only in selected cases, such as for women who have experienced recurrent complications despite aspirin treatment (aspirin failure).

The findings are clearer for inherited thrombophilias. Most prospective studies found no significant association between inherited thrombophilia and placenta‐mediated complications.^438^, ^439^, ^440^, ^441^, ^442^, ^443^ Furthermore, the TIPPS and FRUIT trials found no benefit of LMWH in women with thrombophilia and a history of placenta‐mediated pregnancy complications.^444^, ^445^ These findings were confirmed by a recent individual patient data meta‐analysis that found no benefit of LMWH in decreasing the risk of recurrence of placenta‐mediated complications, including in women with thrombophilia.^140^ Therefore, there is no indication for routine screening for inherited thrombophilia in women with prior FGR.^446^, ^447^

#### 9.3.4 Preconception counselling and management of future pregnancies

Given the considerable risk of recurrence of FGR, efforts should be made to decrease this risk in future pregnancies.^448^ Modifiable risk factors for FGR such as smoking or poor nutritional status should be identified as early as possible and managed accordingly, as discussed in section 5.5.1.

There is some evidence that administration of aspirin can reduce the risk of FGR. However, as described in section 5.5.2 most available data focused on the prevention of pre‐eclampsia as the primary outcome in women at high risk of pre‐eclampsia, with the prevention of FGR being considered a secondary outcome. Data on the prevention of recurrence of FGR in women with a history of FGR are limited.^449^ Therefore, some recommend that aspirin should be considered in women with past FGR only if they have risk factors for pre‐eclampsia at the time of the next pregnancy.^450^ However, we believe that given the safety of aspirin and the overlap in pathogenesis of pre‐eclampsia and FGR, it is reasonable to recommend aspirin to women with a history of placenta‐mediated FGR in the previous pregnancy, using the same regimen of aspirin used for the prevention of pre‐eclampsia. This recommendation is shared by most professional societies.^134^

Data on the role of LMWH to prevent recurrence of placenta‐mediated complications including FGR are conflicting, and this topic is reviewed in section 5.5.2. Based on available data, LMWH therapy should not be used in women with a past history of FGR except in a research setting.

Given the association of insufficient gestational weight gain with FGR, we recommend monitoring of weight gain and informing women about their target weight gain range, as described in section 5.5.1. Other interventions, such as bed rest or nutritional supplements are of unproven benefit and should not be routinely offered.^451^, ^452^ The risk of recurrence can be further stratified in early pregnancy by means of prenatal screening with biochemical markers (PAPP‐A, beta hCG, alpha‐fetoprotein, and PlGF) as well as by uterine artery Doppler, as described in section 5. Due to the increased risk of recurrence, pregnant women with a history of FGR in a previous pregnancy should be managed in a high‐risk pregnancy clinic and should receive closer antenatal surveillance, including close monitoring of fetal growth and maternal blood pressure.^453^

### 9.4 Recommendations

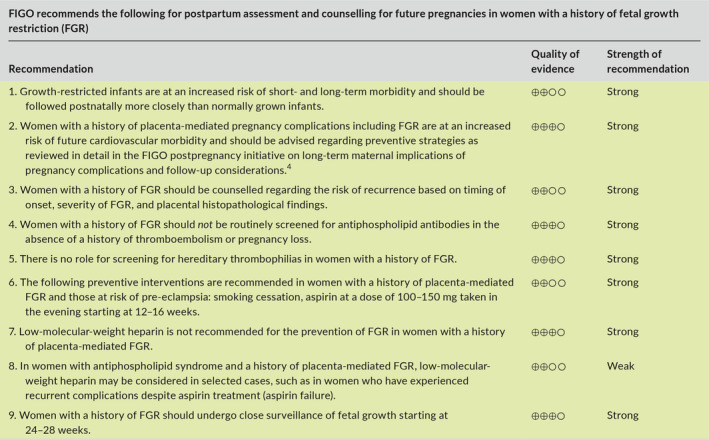

## 10 SUMMARY AND FUTURE RESEARCH DIRECTIONS

FGR is an important cause of stillbirth, neonatal mortality, and short‐ and long‐term neonatal morbidity. Early prediction and preventive strategies, timely diagnosis, and management using a standardized protocol to determine the proper monitoring and timing of delivery can decrease the risk of stillbirth and improve perinatal outcomes in pregnancies complicated by FGR.

Future research should focus on the development of new fetal assessment tools that may improve the accuracy of the prediction of fetal deterioration and thus further optimize timing of delivery of FGR fetuses, as well as on novel treatments that may improve placental function in cases of placenta‐mediated FGR and thereby deferring delivery in cases of early‐onset FGR.

## Supporting information

Table S1‐S2
