# Supplementary material for: FIGO (International Federation of Gynecology and Obstetrics) initiative on fetal growth: Best practice advice for screening, diagnosis, and management of fetal growth restriction
Source: Int J Gynaecol Obstet. 2021 Mar 19;152(Suppl 1):3–57. doi: 10.1002/ijgo.13522 (PMC8252743; doi:10.1002/ijgo.13522)
Supplement: Supplementary file 1 — Table S1‐S2 [file IJGO-152-3-s001.docx]

**SUPPORTING INFORMATION**

**Table S1. Interpretation of strong and conditional (weak) recommendations according to GRADE.^a,b^**

| Implications | 1=Strong recommendation phrased as “we recommend” | 2=Conditional (weak) recommendation phrased as “we suggest” |
| --- | --- | --- |
| For patients | Nearly all patients in this situation would accept the recommended course of action. Formal decision aids are not needed to help patients make decisions consistent with their values and preferences | Most patients in this situation would accept the suggested course of action |
| For clinicians | According to the guidelines, performance of the recommended action could be used as a quality criterion or performance indicator | Decision aids may help patients make a management decision consistent with their values and preferences |
| For policy makers | The recommendation can be adapted as policy in most situations | Stakeholders need to discuss the suggestion |

^a^ Adapted with permission of the American Thoracic Society. © 2020 American Thoracic Society. Schunemann HJ, Jaeschke R, Cook DJ, et al. An official ATS statement: grading the quality of evidence and strength of recommendations in ATS guidelines and recommendations. Am J Respir Crit Care Med 2006;174:605–614. The American Journal of Respiratory and Critical Care Medicine is an official journal of the American Thoracic Society. Readers are encouraged to read the entire article for the correct context. The authors, editors, and The American Thoracic Society are not responsible for errors or omissions in adaptations.

^b^ Both caregivers and care recipients need to be involved in the decision-making process before adopting recommendations.

**Table S2. Interpretation of quality of evidence levels according to GRADE.^a^**

| **Level of evidence** | **Definition** |
| --- | --- |
| High | We are very confident that the true effect corresponds to that of the estimated effect. |
| Moderate  ○ | We are moderately confident in the estimated effect. The true effect is generally close to the estimated effect, but it may be slightly different. |
| Low  ○○ | Our confidence in the estimated effect is limited. The true effect could be substantially different from the estimated effect. |
| Very low  ○○○ | We have very little confidence in the estimated effect. The true effect is likely to be substantially different from the estimated effect. |

^a^Adapted with permission from Balshem et al. [7]. © 2011 Elsevier.
